# Transcriptional upregulation of c‐MYC by AXL confers epirubicin resistance in esophageal adenocarcinoma

**DOI:** 10.1002/1878-0261.12395

**Published:** 2018-11-05

**Authors:** Jun Hong, Selma Maacha, Abbes Belkhiri

**Affiliations:** ^1^ Department of Surgery Vanderbilt University Medical Center Nashville TN USA

**Keywords:** AXL, c‐MYC, epirubicin, esophageal adenocarcinoma, R428, β‐catenin

## Abstract

AXL receptor tyrosine kinase is overexpressed in esophageal adenocarcinoma (EAC) and several other types of malignancies; hence, it may be a valuable therapeutic target. Herein, we investigated the role of AXL in regulating c‐MYC expression and resistance to the chemotherapeutic agent epirubicin in EAC. Using *in vitro* EAC cell models, we found that AXL overexpression enhances epirubicin resistance in sensitive cells. Conversely, genetic knockdown or pharmacological inhibition of AXL sensitizes resistant cells to epirubicin. Notably, we showed that inhibition or knockdown of c‐MYC markedly sensitizes AXL‐dependent resistant cells to epirubicin, and our data demonstrated that AXL promotes epirubicin resistance through transcriptional upregulation of *c‐MYC*. We showed that AXL overexpression significantly increased transcriptional activity, mRNA, and protein levels of c‐MYC. Conversely, AXL knockdown reversed these effects. Mechanistic investigations indicated that AXL upregulates c‐MYC expression through activation of the AKT/β‐catenin signaling pathway. Data from a tumor xenograft mouse model indicated that inhibition of AXL with R428 in combination with epirubicin synergistically suppresses tumor growth and proliferation. Our results demonstrate that AXL promotes epirubicin resistance through transcriptional upregulation of *c‐MYC* in EAC. Our findings support future clinical trials to assess the therapeutic potential of R428 in epirubicin‐resistant tumors with overexpression of AXL and activation of c‐MYC.

**Abbreviations**
DAPI: 4′,6‐diamidino‐2‐phenylindole
EAC: esophageal adenocarcinoma
FACS: fluorescence‐activated cell sorting
FITC: fluorescein isothiocyanate
MTT: 3‐(4,5‐dimethylthiazol‐2‐yl)‐2,5‐diphenyltetrazolium bromide

## 1 Introduction

The incidence rates of esophageal adenocarcinoma (EAC) have been increasing at epidemic proportions in the Western world over the recent decades, reflecting increases in gastroesophageal reflux disease (GERD) and Barrett's esophagus, a precancerous lesion. These risk factors have been related primarily to obesity, poor diet, and the absence of *Helicobacter pylori* infection (reviewed in refs. Jemal *et al*., 2010; Torre *et al*., 2016). The treatment options for EAC includes surgical resection, radiotherapy, and chemotherapy (Ku and Ilson, 2009). Notably, the prognosis of this malignancy remains very poor as the overall 5‐year survival rate of EAC patients is below 20% (reviewed in Ref. Ilson, 2008).

Epirubicin (4′‐epidoxorubicin) is an anticancer chemotherapy drug of the anthracycline class (Bonfante *et al*., 1980). Similar to other anthracyclines, epirubicin acts by intercalating into DNA, binding to DNA topoisomerase II, and leading to DNA synthesis inhibition and DNA cleavage, which ultimately results in cell death (Wang *et al*., 2001). Alone or in combination with other chemotherapy drugs (cisplatin, oxaliplatin, docetaxel, or fluorouracil), epirubicin is widely used in the treatment of gastroesophageal adenocarcinoma (Di Lauro *et al*., 2009; Personeni *et al*., 2016; Pluschnig *et al*., 2013; Webb *et al*., 1997) and other types of malignancies (Pasello *et al*., 2015; Sayal *et al*., 2015). Unfortunately, chemotherapy resistance, whether intrinsic or acquired during treatment, is believed to be the primary cause for treatment failure in the majority of patients with metastatic cancer (Longley and Johnston, 2005). Therefore, understanding of the molecular mechanisms that mediate epirubicin resistance is critical for the development of more effective chemotherapeutic strategies for treatment of EAC.

AXL is a tyrosine kinase receptor (RTK), a member of the TAM family that also includes TYRO 3 and MER, was initially isolated as an oncogene from chronic myelogenous leukemia (O'Bryan *et al*., 1991). Overexpression of AXL is associated with several aspects of cancer such as proliferation, epithelial–mesenchymal transition (EMT), invasion, and resistance to drug treatment (Antony *et al*., 2016; Goruppi *et al*., 1997; Hafizi and Dahlback, 2006; Sainaghi *et al*., 2005). A previous report indicated that the increasing overexpression of AXL was associated with Barrett's carcinogenesis, and could be used as an adverse prognostic marker in EAC (Hector *et al*., 2010). The increasing knowledge on the role of AXL in regulating several aspects of cancer has justified a growing interest in the development and clinical testing of AXL inhibitors for targeted therapies (reviewed in Brown *et al*., 2016; Gay *et al*., 2017; Levin *et al*., 2016). An early study demonstrated that AXL mediates the acquired resistance to EGFR‐targeted therapy in lung cancer (Zhang *et al*., 2012). We have previously reported that AXL plays an important role in tumor necrosis factor‐related apoptosis‐inducing ligand (TRAIL) and cisplatin resistance in EAC cells (Hong and Belkhiri, 2013; Hong *et al*., 2013). However, the mechanisms of AXL in mediating the resistance to other chemotherapeutic drugs have not been fully investigated.

c‐MYC is a basic helix‐loop‐helix leucine zipper transcription factor that plays an essential role in cell cycle progression (Singh and Dalton, 2009), apoptosis (Sheth *et al*., 2014), cellular differentiation (Gomez‐Casares *et al*., 2013), tumorigenesis (Hoffman *et al*., 2002), and drug resistance (Pan *et al*., 2014). Notably, two independent studies have shown that c‐MYC promotes drug resistance by regulating the stemness of a subset of self‐renewing leukemia stem cells, which are thought to contribute to recurrence and treatment failure of acute myeloid leukemia (Li *et al*., 2014; Zhang *et al*., 2015). Frequent gene amplification and overexpression of c‐MYC have been found in invasive EAC (Miller *et al*., 2003; von Rahden *et al*., 2006; Sarbia *et al*., 2001). However, the role of c‐MYC in mediating drug resistance in EAC remains unclear.

In this study, we investigated the implication of AXL in epirubicin resistance in EAC and identified a novel molecular mechanism that controls this effect. We demonstrated that AXL transcriptionally upregulates c‐MYC expression through activation of the AKT/β‐catenin pathway, thereby promoting resistance to epirubicin. We provided evidence that targeting AXL with a specific inhibitor could be an effective treatment strategy to sensitize refractory tumors to chemotherapy.

## 2 Materials and methods

### 2.1 Cell lines and reagents

The human EAC cancer cell lines, OE33, SK‐GT‐4, ESO26, ESO51, OAC M5.1, and OE19, were purchased from Sigma‐Aldrich (St. Louis, MO, USA). FLO‐1 cells were a kind gift from D. Beer (University of Michigan, Ann Arbor, MI, USA). The immortalized Barrett's cells, CP‐A, were obtained from ATCC (Manassas, VA, USA). FLO‐1 and SK‐GT‐4 cells were maintained in Dulbecco's modified Eagle's medium (DMEM; GIBCO, Carlsbad, CA, USA) supplemented with 5% fetal bovine serum (FBS; Invitrogen Life Technologies, Carlsbad, CA, USA) and 1% penicillin/streptomycin (GIBCO). OE33, ESO26, ESO51, OAC M5.1, and OE19 cells were maintained in RPMI medium (GIBCO) supplemented with 5% FBS and 1% penicillin/streptomycin. CP‐A cells were cultured in DMEM/F12 medium supplemented with 0.4 μg·mL^−1^ hydrocortisone, 20 ng·mL^−1^ recombinant human epidermal growth factor, 20 mg·L^−1^ adenine, 140 μg·mL^−1^ bovine pituitary extract, 0.1% ITS Supplement (Sigma‐Aldrich; I1884), 4 mm glutamine, and 5% FBS. During the period of this study, all cell lines were authenticated (once every 6 months) with short tandem repeat (STR) profiling (Genetica DNA Laboratories, Burlington, NC, USA) and confirmed free of mycoplasma (once every 3 months) as determined by MycoSEQ Mycoplasma Real‐Time PCR Detection Kit (Applied Biosystems, Foster City, CA, USA). The AXL inhibitor, R428 (BGB324) was purchased from Selleck Chemicals (Houston, TX, USA). The c‐MYC inhibitor, 10058‐F4, was obtained from Medchem Express (Monmouth Junction, NJ, USA). AXL, PARP, caspase‐3, cleaved caspase‐3, Ki‐67, p‐AKT (S473), AKT, p‐GSK3β (S9), and GSK3β antibodies were purchased from Cell Signaling Technology (Danvers, MA, USA). The c‐MYC antibody was obtained from Santa Cruz Biotechnology (Santa Cruz, CA, USA) and Actin antibody from Sigma‐Aldrich. The p‐AXL (Y779) Antibody was purchased from R&D Systems (Minneapolis, MN, USA). Mouse or rabbit secondary antibodies were obtained from Promega Corporation (Madison, WI, USA).

### 2.2 AXL stable expression

The mammalian expression plasmids of pcDNA4/AXL‐Myc‐His and pcDNA4, kindly provided by R. M. Melillo (University of Naples, Italy), were used to generate stable expression cell lines. OE33 cells, with low endogenous AXL expression, were stably transfected using lipofectamine 2000 (Invitrogen) and subjected to selection with 100 μg·mL^−1^ zeocin (Invitrogen) following standard protocols previously described (Belkhiri *et al*., 2005; Hong *et al*., 2013).

### 2.3 c‐MYC stable expression

The retroviral expression plasmids of pBabe/c‐Myc and control empty vector were kindly provided by W. P. Tansey (Vanderbilt University). Cells were stably transfected using lipofectamine 2000 (Invitrogen) and subjected to selection with 150 μg·mL^−1^ hygromycin (Invitrogen) following standard protocols.

### 2.4 Gene silencing by short interfering RNA

For stable AXL expression silencing, control shRNA or a pool of five validated AXL shRNA lentivirus particles (Sigma‐Aldrich) were used to transduce FLO‐1 and SK‐GT‐4 cells, followed by selection with 1 μg·mL^−1^ puromycin (Invitrogen) for 10 days. The stable c‐MYC expression silencing was achieved by transfection of control shRNA or pRetrosuper/Myc shRNA into FLO‐1 cells by using lipofectamine 2000 (Invitrogen). pRetrosuper/Myc shRNA was a kind gift from Martin Eilers (Addgene plasmid # 15662, Popov *et al*., 2007). Two days after transfection, cells were selected in the presence of 1 μg·mL^−1^ puromycin for 10 days.

### 2.5 Luciferase reporter assays

The *4XEMS‐luc* reporter (a kind gift from S. R. Hann, Vanderbilt University School of Medicine) was used to measure the *c‐MYC* transcriptional activity. Briefly, 25 000 cells per well were seeded into 24‐well plates and were allowed to grow for 24 h. About 250 ng of 4XEMS‐luc reporter plasmid and 100 ng of β‐galactosidase plasmid were then transiently transfected into cells using Lipofectamine 2000 (Invitrogen). About 24 h after transfection, cells were lysed in Reporter Lysis Buffer and luciferase activity was measured using luciferase assay kit (Promega) according to the manufacturer's instructions. Relative Luciferase activities were normalized to β‐galactosidase levels. To assess the transcriptional activity of the β‐catenin/TCF, pTOP‐Flash luciferase reporter, with six TCF binding sites, and its mutant luciferase reporter, pFOP‐Flash, were used. The mutant β‐catenin (S37A), a constitutively active form of β‐catenin, was used as a positive control for pTOP‐Flash reporter as described previously (Vangamudi *et al*., 2011). Relative Luciferase activities were normalized to β‐galactosidase levels.

### 2.6 MTT assay

MTT assay was used to measure cell viability in response to drug treatment. Briefly, 5000 cells per well were seeded into 96‐well plates. After 24 h, the medium was replaced with fresh medium containing various concentrations of drug followed by continued culture in incubator overnight. About 10 μL MTT solution (Sigma‐Aldrich) in PBS (5 mg·mL^−1^) was added to each well followed by 4‐h incubation. Medium was then removed from the plates and 100 μL MTT Solubilization Solution (isopropanol containing 10% Triton X‐100 plus and 0.1 N HCl) was added to each well to dissolve the formazan crystals. The absorbance at 540 nm was measured using a FLUO Star OPTIMA microplate reader (BMG, Cary, NC, USA). All experiments were performed in triplicates and repeated at least three times. Values of IC50, which is the drug concentration required to decrease cell viability by 50%, were obtained from graphpad prism 3.0 Software (GraphPad Software, San Diego, CA, USA) by nonlinear regression analysis with sigmoidal dose‐response equation and are shown as the calculated mean ± SEM.

### 2.7 Clonogenic cell survival assay

Cells were seeded in 6‐well plates at 2000 cells per well. After 24 h, culture medium was replaced with fresh medium containing different concentrations of drug. The next day, cells were washed with PBS and were incubated in drug‐free culture medium for a week. Subsequently, the medium was removed, and cells were fixed with 4% paraformaldehyde solution for 15 min. The cells were then washed with PBS and stained overnight with crystal violet (0.05% crystal violet in 50% methanol). Next day, excess dye was washed with PBS and plates were allowed to dry in air. Images were photographed, and the mean intensity of each image was acquired by using imagej software (https://imagej.nih.gov/ij/). Each experimental condition was assayed in triplicate.

### 2.8 Apoptosis assay

Cells were seeded in 6‐well plates at a density of 10^5^ cells per well. The next day, cells were treated with vehicle or epirubicin for 24 h. Cells were then harvested and stained with Annexin‐V fluorescein isothiocyanate (FITC; Trevigen, Gaithersburg, MD, USA) and 4,6‐Diamidino‐2‐phenylindole dihydrochloride (DAPI; Sigma‐Aldrich). The samples were then subjected to fluorescence‐activated cell sorting (FACS) analysis by a flow cytometer (Becton Dickinson, Franklin Lakes, NJ, USA). Apoptotic cell death was determined by counting cells that stained positive for Annexin‐V FITC and negative for DAPI (early apoptosis) in addition to cells that are positive for both Annexin‐V FITC and DAPI (late apoptosis).

### 2.9 Quantitative real‐time RT‐PCR

Total RNA was extracted from cells using Trizol (Invitrogen), and cDNA was synthesized using SensiFAST™ cDNA Synthesis Kit (Bioline, Taunton, MA, USA). Synthesized cDNA was diluted into 100 μL, and 2 μL of this dilution was used for quantitative real‐time reverse transcriptase PCR (qRT‐PCR). The qRT‐PCR was performed with a Bio‐Rad CFX Connect Real‐time System in a 10‐μL reaction volume using iQ SYBR Green Supermix (Bio‐Rad Laboratories Inc., Hercules, CA, USA) with the gene‐specific primers (Table S1). The threshold cycle number was determined by cfx manager™ software version 3.0 (Bio‐Rad). Reactions were performed in triplicate, and the threshold cycle numbers were averaged. The data were normalized to the *HPRT1* housekeeping gene. The relative mRNA expression levels were calculated according to the formula 2^(RT − ET)^/2^(Rn − En)^, as described previously (Dematteo *et al*., 2002).

### 2.10 *c‐MYC* mRNA decay analysis

Cells were treated with Actinomycin D at a final concentration of 2 μg·mL^−1^ and harvested at 0‐, 10‐, 20‐, 30‐, and 60‐min time points. Total RNA was extracted, and cDNA was synthesized. Relative mRNA expression of *c‐MYC* was determined by qRT‐PCR with specific primers (Table S1) at the indicated time points. The threshold cycle numbers were normalized to β‐actin housekeeping gene. The mRNA degradation curve was generated by plotting the relative expression values as a function of the time period of Actinomycin D treatment. Linear regression was carried out and the mRNA half‐life (*t*_1/2_), which represents the time for degradation of 50% of the mRNA, was calculated from the fitted line equation.

### 2.11 Western blot analysis

Cell was scraped and centrifuged at 4 °C. Pellets were lysed in RIPA Lysis Buffer System supplemented with 1 × Halt protease inhibitor cocktail and 1 × Halt phosphatase inhibitor cocktail (Santa Cruz Biotechnology). Protein concentrations were measured using Bio‐Rad protein Assay (Bio‐Rad). Proteins were separated by sodium dodecyl sulfate polyacrylamide gel electrophoresis (SDS/PAGE) and transferred to Protran nitrocellulose membranes (Whatman, Boston, MA, USA). Membranes were probed with specific primary antibodies, followed by horseradish peroxidase (HRP)‐conjugated secondary antibodies (Promega). Protein bands were visualized using Amersham ECL western blotting detection reagent (GE Healthcare, Pittsburgh, PA, USA), and images were taken on the ChemiDoc XRS System (Bio‐Rad). The protein bands intensities were semiquantitatively analyzed by densitometry using imagej software (NIH Image).

### 2.12 c‐MYC protein stability assay

Cells were treated with 80 μg·mL^−1^ of cycloheximide (CHX) and harvested at 0‐, 10‐, 20‐, 30‐, and 60‐min time points. Proteins were extracted and analyzed by western blotting to assess c‐MYC protein stability. The protein bands intensities were semiquantitatively analyzed by densitometry using imagej software (NIH Image). c‐MYC band intensities per treatment condition were normalized to β‐actin. The protein degradation curve was generated by plotting band intensities ratios as a function of the time period of CHX treatment. Linear regression was performed and the protein half‐life (*t*_1/2_), which is expressed as the time for degradation of 50% of the protein, was calculated from the fitted line equation.

### 2.13 *In vivo* tumor xenograft mouse model

Four‐week‐old B6;129‐Rag2tm1FwaII2rgtm1Rsky/DwlHsd (R2G2) female mice were purchased from Envigo RMS Division (Indianapolis, IN, USA) and were maintained under specific pathogen‐free conditions. The mice were randomized into four groups (12 xenografts each group). FLO‐1 cells (5 × 10^6^) suspended in 200 μL DMEM/growth factor‐reduced Matrigel (BD Biosciences, San Jose, CA, USA) mixture (50% DMEM supplemented with 10% FBS and 50% Matrigel) were injected subcutaneously into the flank regions of the mice. The tumors were allowed to grow until 500 mm^3^ in size (approximately 30 days from injection) before starting single or combined treatments for 10 days. Epirubicin was administrated by i.p. injection once every other day at a dose of 5 mg·kg^−1^. R428 was formulated in 0.5% hydroxypropylmethylcellulose with 0.1% Tween 80 and was administered by oral gavage twice a day at a dose of 10 mg·kg^−1^. To determine the tumor xenograft volume, the greatest longitudinal diameter (length) and the greatest transverse diameter (width) were serially measured every alternate day by external caliper. Tumor volume was calculated by the following formula: Tumor volume = 1/2 (length ×width^2^). At the end of treatments, the xenografts were isolated from control and treatment groups and subjected to H&E staining and immunohistochemistry using p‐AXL (Y779), Ki‐67, and cleaved caspase‐3 antibodies. The animal protocol was approved by the Vanderbilt Institutional Animal Care and Use Committee.

### 2.14 Immunohistochemistry

After completion of mouse treatments, the xenograft tumors were isolated, fixed in formalin, and paraffin‐embedded. Tissue sections (4 μm) were deparaffinized in xylene and rehydrated via graded ethanol. The sections were subjected to heat‐induced antigen retrieval in sodium citrate buffer (10 mm, pH 6) at 104 °C for 20 min, and treated with H_2_O_2_ (0.02%) for 10 min to inactivate endogenous peroxidases. The sections were blocked with Dako Ready‐to‐use Protein Block Serum‐Free (X0909; Dako North America, Inc., Carpinteria, CA, USA) for 15 min, and then incubated overnight with p‐AXL (Y799; 1 : 200 dilution), Ki‐67 (1 : 200 dilution), or cleaved caspase‐3 (1 : 400 dilution) primary antibodies. Next, the sections were incubated with Dako EnVision+ System‐HRP labeled Polymer (K4002; Dako North America, Inc.) for 30 min, followed by the application of 3, 3′‐diaminobenzidine (DAB) for 5 min, and counterstaining of the tissues with hematoxylin. Images were acquired by using an Olympus BX51 microscope (Olympus Co., Center Valley, PA, USA). The protein expression level of p‐AXL (Y779) was determined by using the IHC toolbox plugin in imagej software (https://imagej.nih.gov/ij/plugins/ihc-toolbox/index.html). Expression levels of Ki‐67 or cleaved caspase‐3 were reported as % of positive cells relative to total cell number in xenografts from four groups of mice.

### 2.15 Statistical analysis

The results from at least three independent experiments are shown as mean ± SEM. Differences were analyzed by Student's *t*‐test or one‐way ANOVA followed by the Newman–Keuls *post hoc* test. All the statistical analyses were performed using the graphpad prism, version 5.0 (GraphPad Software). Differences with *P* values ≤ 0.05 are considered significant.

## 3 Results

### 3.1 AXL expression promotes epirubicin resistance in esophageal adenocarcinoma cells

**Figure 1:**
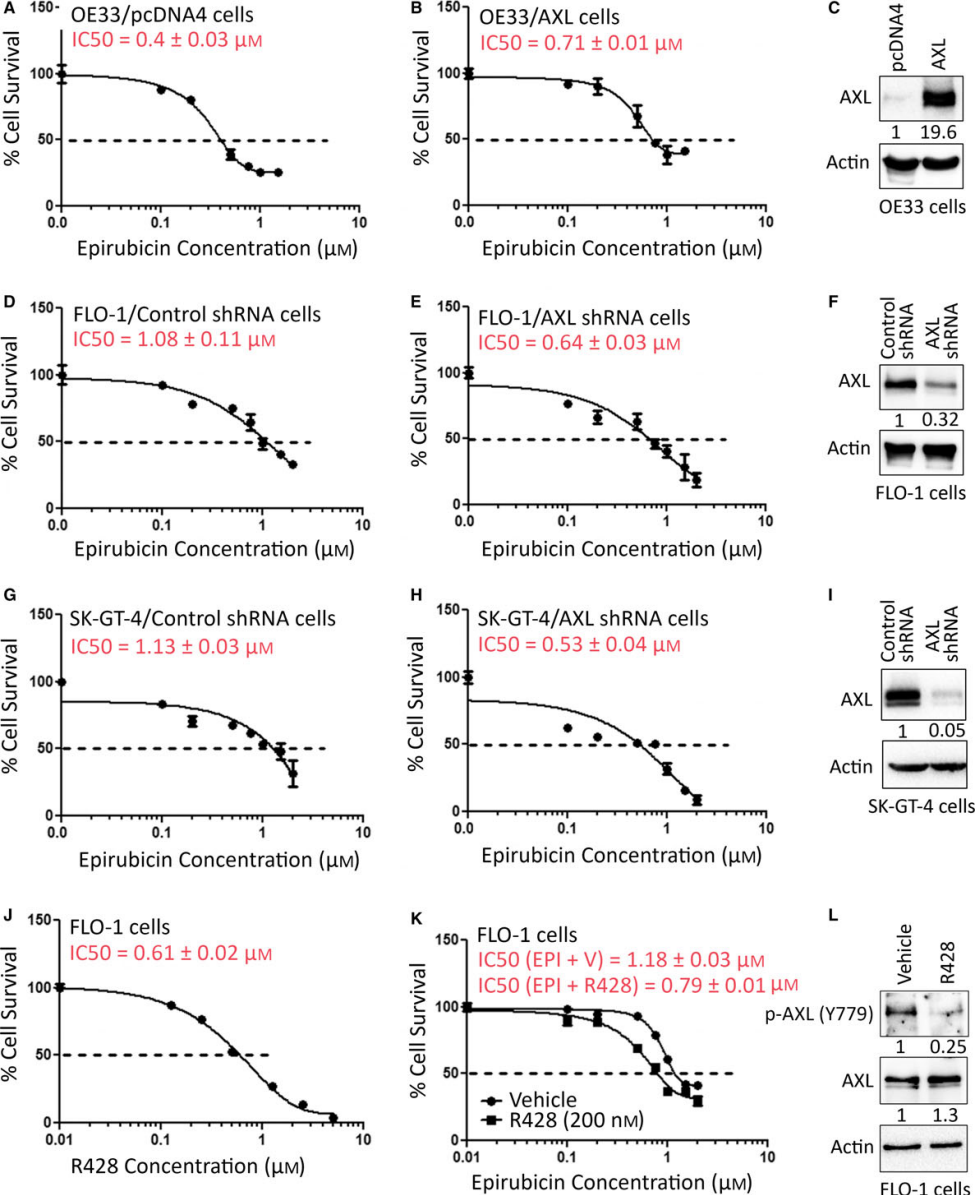
Modulation of AXL expression regulates epirubicin resistance. OE33/pcDNA4 (A), OE33/AXL (B), FLO‐1/Control shRNA (D), FLO‐1/AXL shRNA (E), SK‐GT‐4/Control shRNA (G), and SK‐GT‐4/AXL shRNA (H) stable cells were treated with vehicle or with the indicated concentrations of epirubicin for 24 h. Protein expression levels of AXL in OE33 (C), FLO‐1 (F), and SK‐GT‐4 (I) cell models were confirmed by western blot analysis. Cell viability was evaluated by MTT assay. (J) FLO‐1 cells were treated with vehicle or with the indicated concentrations of R428 (an inhibitor of AXL) for 24 h. Cell viability was determined by MTT assay. (K) FLO‐1 cells were treated with increasing concentrations of epirubicin in combination with vehicle or a low concentration of R428 (200 nm) for 24 h. Cell viability was assessed by MTT assay. (L) The inhibitory effect of R428 (200 nm) on AXL kinase activity in FLO‐1 cells was confirmed by western blot analysis of p‐AXL (Y779) protein. Gel loading was normalized for equal β‐actin. Results from three independent experiments are represented as mean ± SEM.

Epirubicin alone or in combination with other chemotherapeutic drugs has been used as a first‐line therapy in patients with upper gastrointestinal adenocarcinoma. Unfortunately, resistance to epirubicin is a challenging clinical problem and understanding the underlying mechanism is of major importance in overcoming the drug resistance. We first investigated if there was an association between AXL expression and resistance to epirubicin in EAC cells. Western blot analysis of AXL protein expression in a panel of 7 EAC cell lines indicated high expression in FLO‐1 and SK‐GT‐4, and very low expression in ESO26, OAC M5.1, ESO51, OE33, and OE19 (Fig. S1A). We next investigated whether high AXL expression correlated with resistance to epirubicin in EAC cell lines. Indeed, the MTT cell viability assay data showed that high AXL‐expressing cells, SK‐GT‐4 (IC_50_ = 1.12 ±0.01 μm, Fig. S1C) and FLO‐1 (IC_50_ = 1.13 ±0.07 μm, Fig. S1D), exhibited higher epirubicin resistance than low AXL‐expressing cells, OE33 (IC_50_ =0.46 ± 0.03 μm, Fig. S1B). To ascertain the role of AXL expression in regulating epirubicin resistance, we subjected OE33 cells stably expressing AXL or empty vector to the MTT cell viability assay after treatment with various concentrations of epirubicin for 24 h. The data indicated that the overexpression of AXL in OE33 cells doubled cell viability relative to control cells in response to epirubicin (Fig. 1A,B). In fact, the epirubicin IC_50_ increased from 0.4 ± 0.03 μm in OE33/pcDNA4 cells (Fig. 1A) to 0.71 ± 0.01 μm in OE33/AXL cells (Fig. 1B). The AXL protein expression in OE33 cells was verified by western blot analysis (Fig. 1C). Conversely, the knockdown of endogenous AXL in FLO‐1 and SK‐GT‐4 cells enhanced the sensitivity to epirubicin (Fig. 1D,E,G,H). The results indicated that the epirubicin IC_50_ was 1.08 ± 0.11 μm in FLO‐1/Control shRNA cells (Fig. 1D), 0.64 ± 0.03 μm in FLO‐1/AXL shRNA cells (Fig. 1E), 1.13 ± 0.03 μm in SK‐GT‐4/Control shRNA cells (Fig. 1G), and 0.53 ± 0.04 μm in SK‐GT‐4/AXL shRNA cells (Fig. 1H). The AXL protein expression in FLO‐1 cells (Fig. 1F) and SK‐GT‐4 cells (Fig. 1I) was verified by western blot analysis.

To further confirm the prosurvival function of AXL in response to epirubicin, we tested the AXL inhibitor, R428, in FLO‐1 cells. We first treated the cells with increasing concentrations of R428 for 24 h and evaluated cell viability by the MTT cell viability assay. The data indicated that the R428 IC_50_ was 0.61 ± 0.02 μm in FLO‐1 cells (Fig. 1J). We next investigated if inhibition of AXL with a low concentration of R428 (200 nm, a third of IC_50_) could sensitize FLO‐1 cells to epirubicin. The MTT assay data indicated that cell viability was much greater following treatment with epirubicin and vehicle (IC_50_ = 1.18 ± 0.03 μm, Fig. 1K) than the combination treatment with epirubicin and R428 (IC_50_ = 0.79 ± 0.01 μm, Fig. 1K). The inhibitory effect of R428 on AXL was verified by western blot analysis (Fig. 1L). To confirm the short‐term (24 h) survival data, we subjected OE33 cells stably expressing AXL or pcDNA4, and FLO‐1 cells stably expressing AXL shRNA or control shRNA to a long‐term (1 week) clonogenic survival assay. The data showed that the overexpression of AXL in OE33 cells significantly increased cell survival by 23.5% relative to control (*P* < 0.01) in response to epirubicin (Fig. S2A). In contrast, knocking down of endogenous AXL in FLO‐1 cells significantly reduced cell survival by 22% relative to control (*P* < 0.01) in response to epirubicin (Fig. S2B). Together, the data clearly demonstrated that AXL expression positively regulates epirubicin resistance in EAC cells.

### 3.2 AXL mitigates epirubicin‐induced apoptosis and activation of caspase‐3

**Figure 2:**
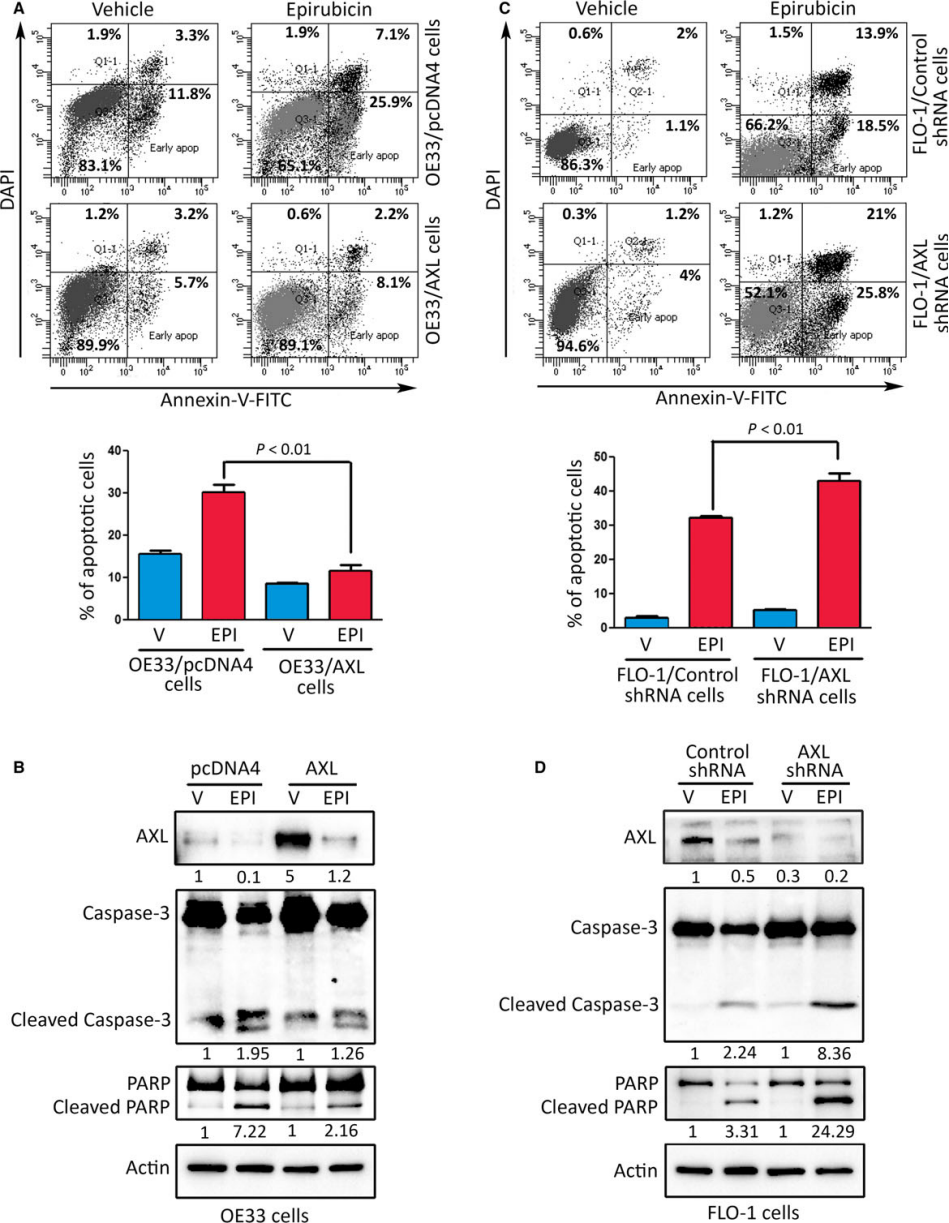
AXL attenuates epirubicin‐induced apoptosis. (A) OE33/pcDNA4 and OE33/AXL stable cells were treated with vehicle or epirubicin (0.5 μm) for 24 h. Apoptosis was determined by Annexin‐V/DAPI staining and FACS analysis. (B) Immunoblot analysis of AXL, caspase‐3, and PARP proteins in OE33/pcDNA4 and OE33/AXL cells after treatment with vehicle or epirubicin as described in panel A. (C) Apoptosis in FLO‐1/control shRNA and FLO‐1/AXL shRNA cells after treatment with vehicle or epirubicin (1 μm) for 24 h was assessed as in panel A. (D) Western blot analysis of AXL, caspase‐3, and PARP proteins in FLO‐1/control shRNA and FLO‐1/AXL shRNA cells after treatment with vehicle or Epirubicin as in panel C. Gel loading was normalized for equal β‐actin. Results from at least three independent experiments are represented as mean ± SEM. Statistical significance was evaluated by Student's *t*‐test.

We investigated whether the prosurvival function of AXL involves suppression of apoptosis induced by epirubicin. The Annexin‐V/DAPI and FACS analysis data showed that the stable overexpression of AXL in OE33 cells significantly reduced apoptosis events by 68% relative to control in response to epirubicin (*P* < 0.01, Fig. 2A). Accordingly, western blot analysis indicated distinctly less protein levels of cleaved caspase‐3 and PARP in AXL‐expressing cells than control cells after treatment with epirubicin (Fig. 2B). Conversely, the stable knockdown of endogenous AXL in FLO‐1 cells significantly increased apoptosis events by 44% relative to control in response to epirubicin (*P* < 0.01, Fig. 2C). In line with these results, western blot analysis indicated that knockdown of AXL markedly increased protein levels of cleaved caspase‐3 and PARP relative to control in response to epirubicin (Fig. 2D).

### 3.3 AXL promotes epirubicin resistance through upregulation of c‐MYC expression and activity

**Figure 3:**
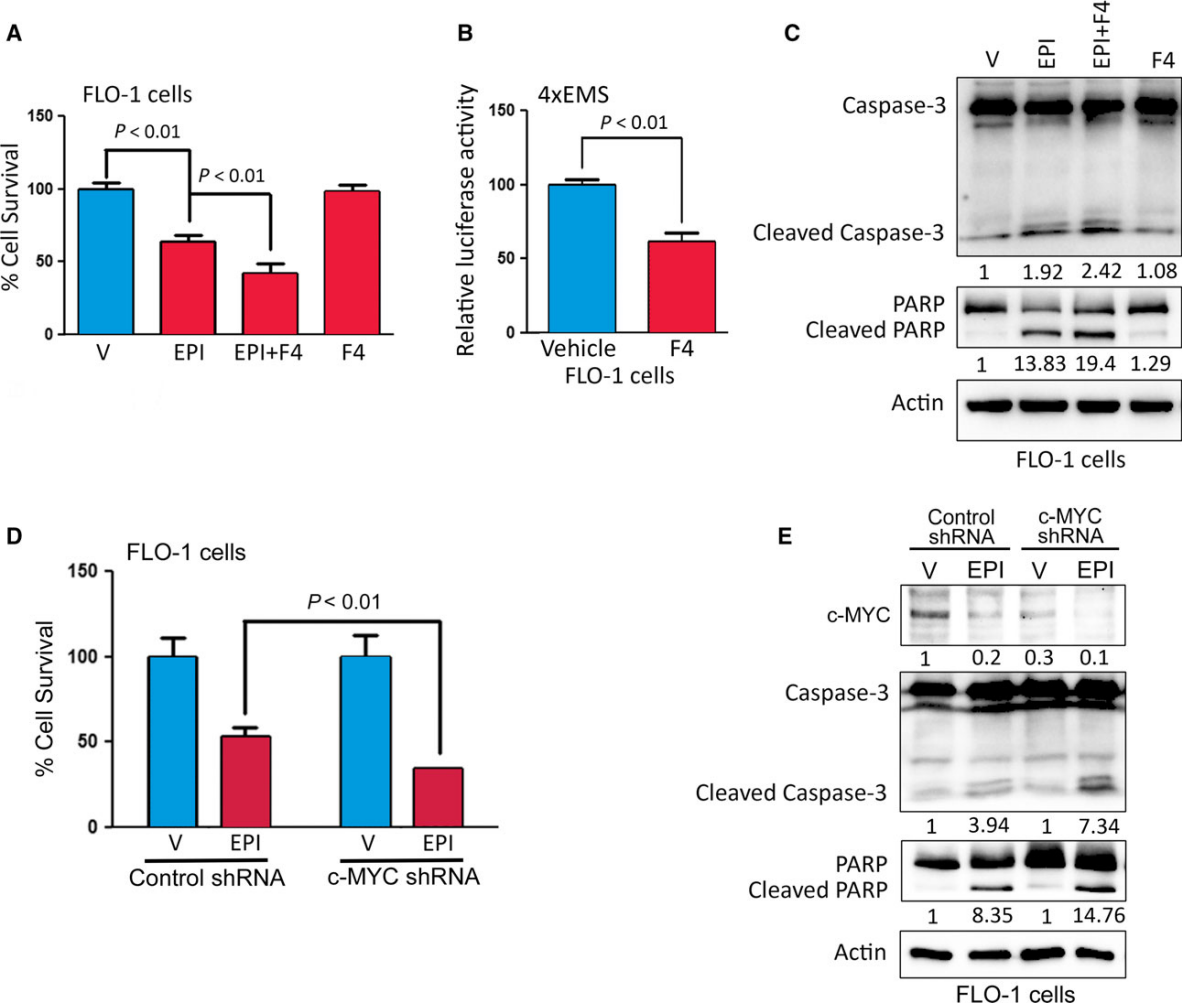
AXL‐mediated epirubicin resistance depends on c‐MYC activity. (A) FLO‐1 cells were treated with vehicle, epirubicin (1 μm) alone, epirubicin (1 μm) in combination with 10058‐F4 (5 μm), or 10058‐F4 (5 μm) alone for 24 h. Cell viability was evaluated by MTT assay. (B) To measure the *c‐MYC* transcriptional activity, FLO‐1 cells were transfected with *4xEMS‐Luc* reporter and β‐galactosidase plasmids, and treated with vehicle or 10058‐F4 (5 μm) for 24 h. (C) Western blot analysis of caspase‐3 and PARP proteins in FLO‐1 cells after treatment with vehicle, epirubicin alone, epirubicin in combination with 10058‐F4, or 10058‐F4 alone as described in panel A. (D) FLO‐1 cells were subjected to Knockdown of c‐MYC by shRNA or control shRNA and treated with vehicle or epirubicin (1 μm) for 24 h. Cell viability was assessed by MTT assay. (E) FLO‐1 cells with control or c‐MYC knockdown and treated with vehicle or epirubicin, as described in panel D, were subjected to western blot analysis of caspase‐3 and PARP proteins. Gel loading was normalized for equal β‐actin. Data from three independent experiments are represented as mean ± SEM. Statistical significance was evaluated by Student's *t*‐test.

As c‐MYC has been shown to play a role in cancer therapy drug resistance (Li *et al*., 2014; Zhang *et al*., 2015), we investigated whether AXL‐dependent epirubicin resistance involves regulation of c‐MYC expression in EAC cells. We first examined *c‐MYC* gene expression in EAC based on the publically available RNAseq data from the Cancer Genome Atlas (TCGA). The dataset comprises 88 EAC primary tumors and 11 normal esophageal tissues. The expression of *c‐MYC* was evaluated relative to its median expression in the normal esophageal tissues and was found to be overexpressed in approximately 68% of the tumors (Fig. S3). Additionally, western blot analysis data showed an overall trend of co‐expression of AXL and c‐MYC proteins in a panel of 7 EAC cell lines, except for ESO26 cells (Fig. S1A). Collectively, these findings suggest that c‐MYC expression could mediate survival and drug resistance in EAC. To test this hypothesis, we treated the resistant FLO‐1 cells with epirubicin (1 μm, IC_50_ dose) alone or in combination with the c‐MYC inhibitor, 10058‐F4 (5 μm, a sub‐lethal dose). The MTT assay data showed that inhibition of c‐MYC significantly decreased cell viability in response to treatment with epirubicin (*P* < 0.01, Fig. 3A). The inhibition of c‐MYC transcriptional activity by 10058‐F4 was confirmed by a luciferase assay using 4xEMS‐luc reporter (Fig. 3B). Consistent with the cell viability data, we found that the combination treatment of FLO‐1 cells with epirubicin and 10058‐F4 induced more apoptosis than the treatment with epirubicin alone, as indicated by western blot analysis of apoptosis markers (Fig. 3C). In fact, the combination treatment led to higher protein levels of cleaved forms of caspase‐3 and PARP than the treatment with epirubicin alone (Fig. 3C). In addition, as indicated by MTT assay data, we found that the knockdown of c‐MYC by shRNA significantly sensitized FLO‐1 cells to epirubicin (*P* < 0.01, Fig. 3D). The western blot analysis data confirmed that the knockdown of c‐MYC increased apoptosis relative to control, as indicated by changes in protein levels of apoptosis markers in response to epirubicin (Fig. 3E). We further confirmed the involvement of c‐MYC in mediating AXL‐dependent epirubicin resistance. Indeed, we found that the reconstitution of exogenous c‐MYC expression in sensitive AXL knockdown FLO‐1 cells significantly restored epirubicin resistance as indicated by MTT data (*P* < 0.01, Fig. S4).

**Figure 4:**
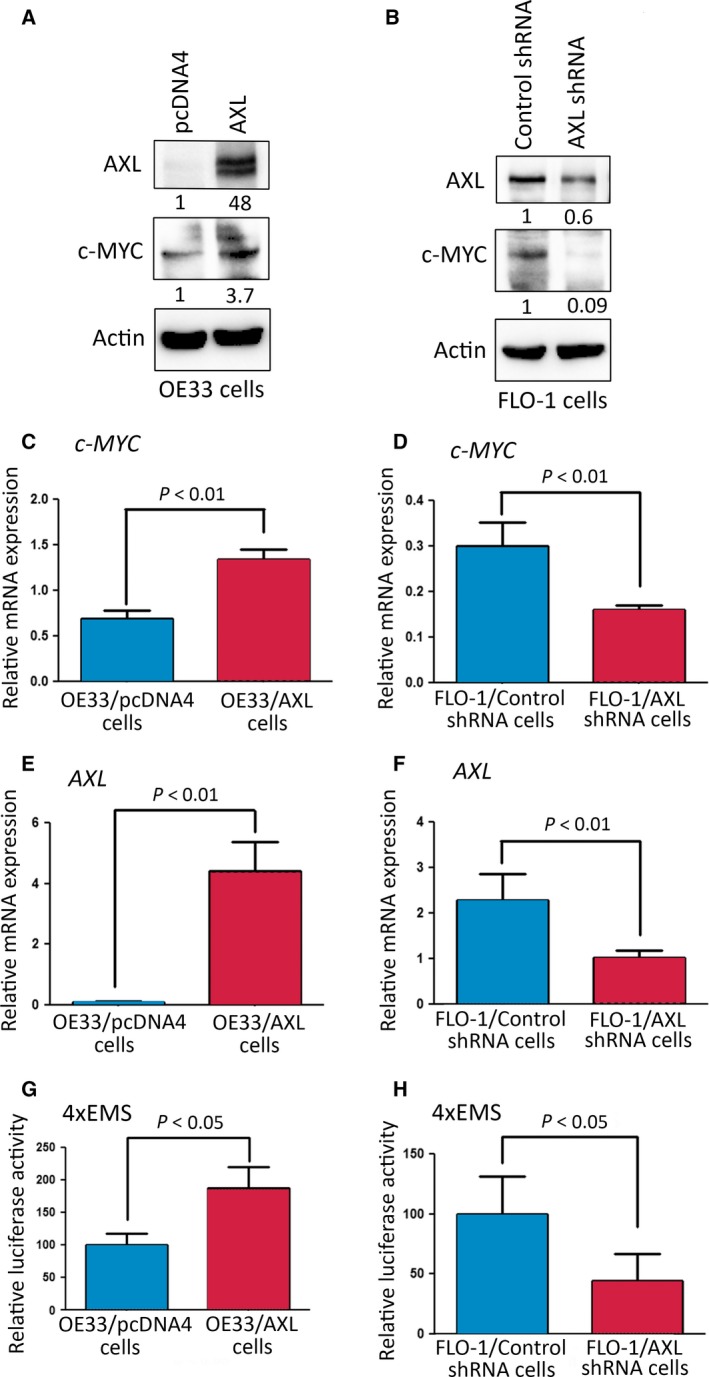
AXL upregulates expression and activity of c‐MYC in EAC cells. Western blot analysis of AXL and c‐MYC proteins in OE33/pcDNA4 and OE33/AXL cells (A) and in FLO‐1/Control shRNA and FLO‐1/AXL shRNA cells (B). Gel loading was normalized for equal β‐actin. Real‐time quantitative PCR analysis of *c‐MYC* and *AXL* in OE33/pcDNA4 and OE33/AXL cells (C, E), and in FLO‐1/Control shRNA and FLO‐1/AXL shRNA cells (D, F). To measure the *c‐MYC* transcriptional activity, OE33/pcDNA4, OE33/AXL, FLO‐1/Control shRNA, and FLO‐1/AXL shRNA cells were transfected with *4xEMS‐Luc* reporter and β‐galactosidase plasmids (G, H). Data from three independent experiments are represented as mean ± SEM. Statistical significance was evaluated by Student's *t*‐test.

We next investigated c‐MYC expression and transcriptional activity following modulation of AXL expression in EAC cells. Western blot analysis data showed that the overexpression of AXL in OE33 cells increased c‐MYC protein level relative to control cells (Fig. 4A). Conversely, knockdown of endogenous AXL in FLO‐1 cells decreased c‐MYC protein level in comparison with control cells (Fig. 4B). The quantitative RT‐PCR data confirmed that the overexpression of AXL in OE33 cells significantly increased *c‐MYC* mRNA level (*P* < 0.01, Fig. 4C), and knockdown of endogenous AXL in FLO‐1 cells significantly decreased *c‐MYC* mRNA level (*P* < 0.01, Fig. 4D) relative to corresponding control cells. The *AXL* mRNA expression in OE33 cells (Fig. 4E) and FLO‐1 cells (Fig. 4F) was verified by quantitative RT‐PCR. Furthermore, the c‐MYC/4xEMS luciferase assay data showed that the *c‐MYC* transcriptional activity was significantly increased after the overexpression of AXL in OE33 cells (*P* < 0.05, Fig. 4G), and significantly decreased following the knockdown of endogenous AXL in FLO‐1 cells (*P* < 0.05, Fig. 4H) relative to corresponding control cells. We next investigated whether AXL regulates *c‐MYC* mRNA stability. We treated OE33/pcDNA4 and OE33/AXL cells with Actinomycin D (2 μg·mL^−1^, an inhibitor of mRNA biosynthesis) for various time points and determined *c‐MYC* relative mRNA expression by quantitative RT‐PCR. The data demonstrated that the rate of *c‐MYC* mRNA degradation was not regulated by AXL, as indicated by the mRNA half‐life in both cell lines (Fig. S5A). The data clearly indicated that AXL expression had no significant effect on *c‐MYC* mRNA stability in EAC cells. Furthermore, we investigated if AXL regulates c‐MYC protein stability. We treated FLO‐1/Control shRNA and FLO‐1/AXL shRNA cells with cycloheximide (80 μg·mL^−1^, an inhibitor of protein synthesis) for different time points and evaluated c‐MYC protein levels by western blot analysis. The data showed that the c‐MYC protein degradation rate was not significantly affected by AXL expression, as indicated by the protein half‐life in both cell lines (Fig. S5B,C). The results demonstrated that AXL transcriptionally upregulates c‐MYC mRNA and protein expression as well as its transcriptional activity in EAC cells. Collectively, the data indicated that c‐MYC expression and activity mediate AXL‐dependent epirubicin resistance in EAC cells.

### 3.4 AXL upregulates c‐MYC expression through activation of AKT/β‐catenin pathway

**Figure 5:**
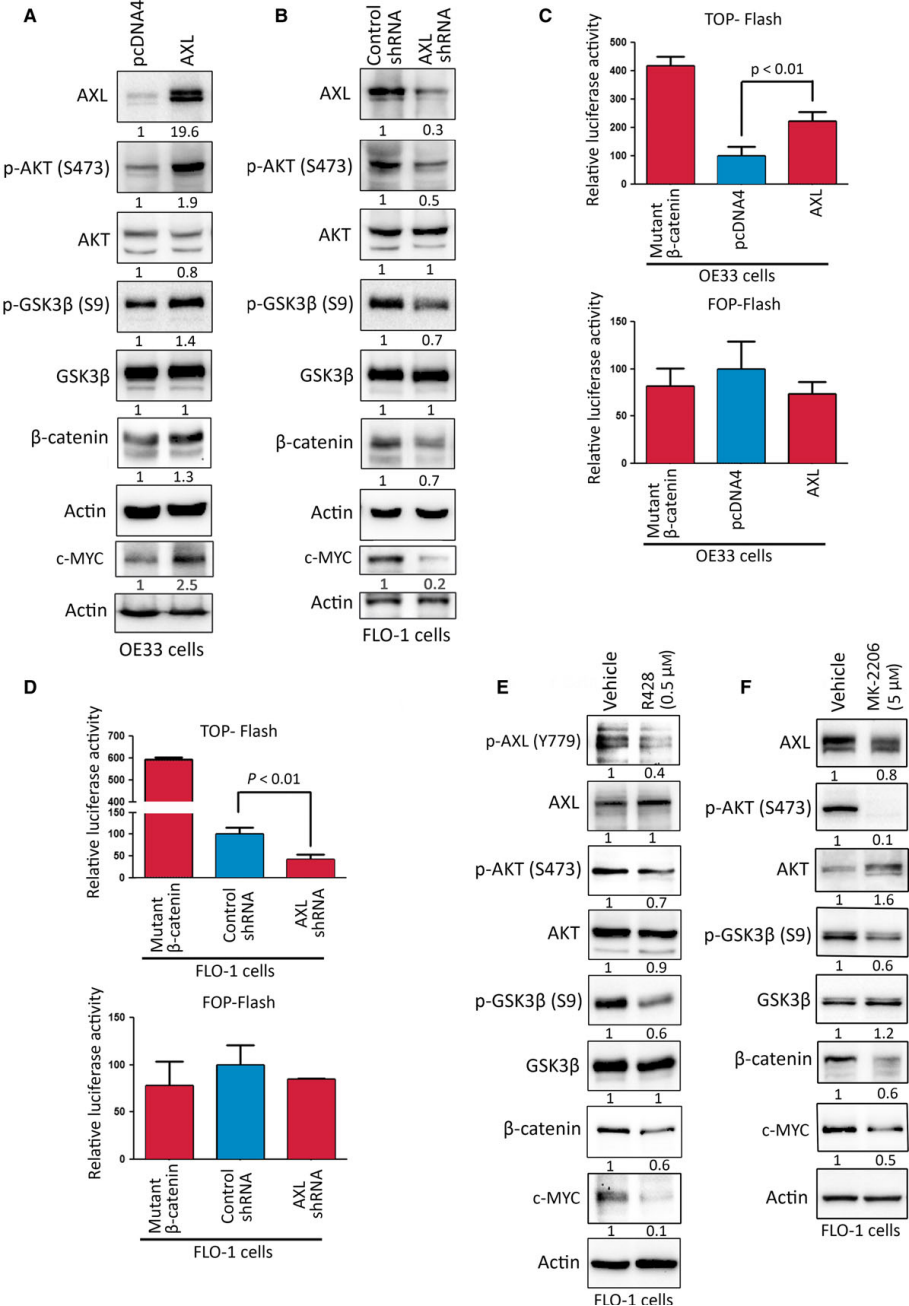
AXL enhances c‐MYC expression through regulation of AKT/β‐catenin pathway. Western blot analysis of AXL, p‐AKT (S473), AKT, p‐GSK3β (S9), GSK3β, β‐catenin, and c‐MYC proteins in OE33/pcDNA4 and OE33/AXL cells (A), and in FLO‐1/Control shRNA and FLO‐1/AXL shRNA cells (B). OE33/pcDNA4, OE33/AXL, FLO‐1/Control shRNA, and FLO‐1/AXL shRNA cells were transfected with pTOP‐Flash or pFOP‐Flash (a negative control) luciferase reporter plasmids to measure the β‐catenin/TCF transcriptional activity (C, D). In a control experiment, OE33 cells (C) and FLO‐1 cells (D) were transfected with the constitutively active mutant β‐catenin (S37A) in combination with pTOP‐Flash or pFOP‐Flash plasmids. (E) FLO‐1 cells were treated with vehicle or R428 (AXL inhibitor, 0.5 μm) for 24 h and subjected to western blot analysis of p‐AXL (Y779), AXL, p‐AKT (S473), AKT, p‐GSK3β (S9), GSK3β, β‐catenin, and c‐MYC proteins. (F) FLO‐1 cells were treated with vehicle or MK‐2206 (AKT inhibitor, 5 μm) for 24 h and analyzed by western blotting for expression of AXL, p‐AKT (S473), AKT, p‐GSK3β (S9), GSK3β, β‐catenin, and c‐MYC proteins. Gel loading was normalized for equal β‐actin. Data from three independent experiments are represented as mean ± SEM. Statistical significance was evaluated by Student's *t*‐test.

Our data indicated that AXL transcriptionally upregulates c‐MYC expression in EAC cells (Fig. 4). Therefore, we investigated whether AXL activates the AKT/β‐catenin signaling, a well‐established pathway in the regulation of c‐MYC expression (Vangamudi *et al*., 2010, 2011). Indeed, western blot data indicated that the overexpression of AXL in OE33 cells increased protein levels of p‐AKT (S473), p‐GSK3β (S9), β‐catenin, and c‐MYC as compared to control cells (Fig. 5A). Conversely, the knockdown of endogenous AXL in FLO‐1 cells decreased p‐AKT (S473), p‐GSK3β (S9), β‐catenin, and c‐MYC protein levels as compared to control cells (Fig. 5B). We next investigated if modulation of AXL expression regulates β‐catenin/TCF transcriptional activity using the luciferase reporter constructs, pTOP‐Flash (with six TCF/LEF binding sites) and pFOP‐Flash (with mutated TCF/LEF binding sites), as a negative control. The data showed that the overexpression of AXL in OE33 cells led to a twofold induction in the pTOP‐Flash reporter activity relative to control (*P* < 0.01, Fig. 5C). In contrast, the knockdown of endogenous AXL in FLO‐1 cells resulted in a twofold decrease in the pTOP‐Flash reporter activity relative to control (*P* < 0.01, Fig. 5D). The specificity of β‐catenin/TCF was confirmed by the expression of the negative control reporter, pFOP‐Flash, as indicated by the insignificant change of luciferase activities in response to modulation of AXL expression in OE33 cells (Fig. 5C, low panel) and FLO‐1 cells (Fig. 5D, low panel). We used the constitutively active mutant β‐catenin (S37A) as a positive control for pTOP‐Flash reporter assay. To further confirm the role of AXL in regulating the AKT/β‐catenin pathway and c‐MYC expression, we treated FLO‐1 cells with vehicle or the AXL inhibitor, R428 (0.5 μm), for 24 h. As expected, western blot data indicated that the inhibition of AXL markedly decreased protein levels of p‐AXL (Y779), p‐AKT (S473), p‐GSK3β (S9), β‐catenin, and c‐MYC (Fig. 5E). Additionally, we showed that pharmacologic inhibition of AKT by MK‐2206 (5 μm) led to a marked decrease in p‐GSK3β (S9), β‐catenin, and c‐MYC protein levels in FLO‐1 cells (Fig. 5F). Together with the data indicating that knocking down (Fig. 5B) or inhibition (Fig. 4E) of AXL reduces p‐AKT (S473) and c‐MYC protein levels in the FLO‐1 cells, we concluded that AXL‐dependent c‐MYC expression is mediated by AKT.

Based on our findings that AKT mediates AXL‐dependent c‐MYC expression (Fig. 5), and AXL‐induced epirubicin resistance requires upregulation of c‐MYC expression (Figs 3 and 4), we next investigated if inhibition of AKT could sensitize FLO‐1 cells to epirubicin. Indeed, the MTT assay data indicated that inhibition of AKT with MK‐2206 (5 μm) significantly decreased cell viability in response to treatment with epirubicin (1 μm,* P* < 0.01, Fig. S6). Collectively, the data clearly demonstrated that AXL upregulates c‐MYC expression through activation of the AKT/β‐catenin signaling pathway in EAC cells.

### 3.5 Targeting AXL with R428 effectively sensitizes xenografted tumors to epirubicin *in vivo*

**Figure 6:**
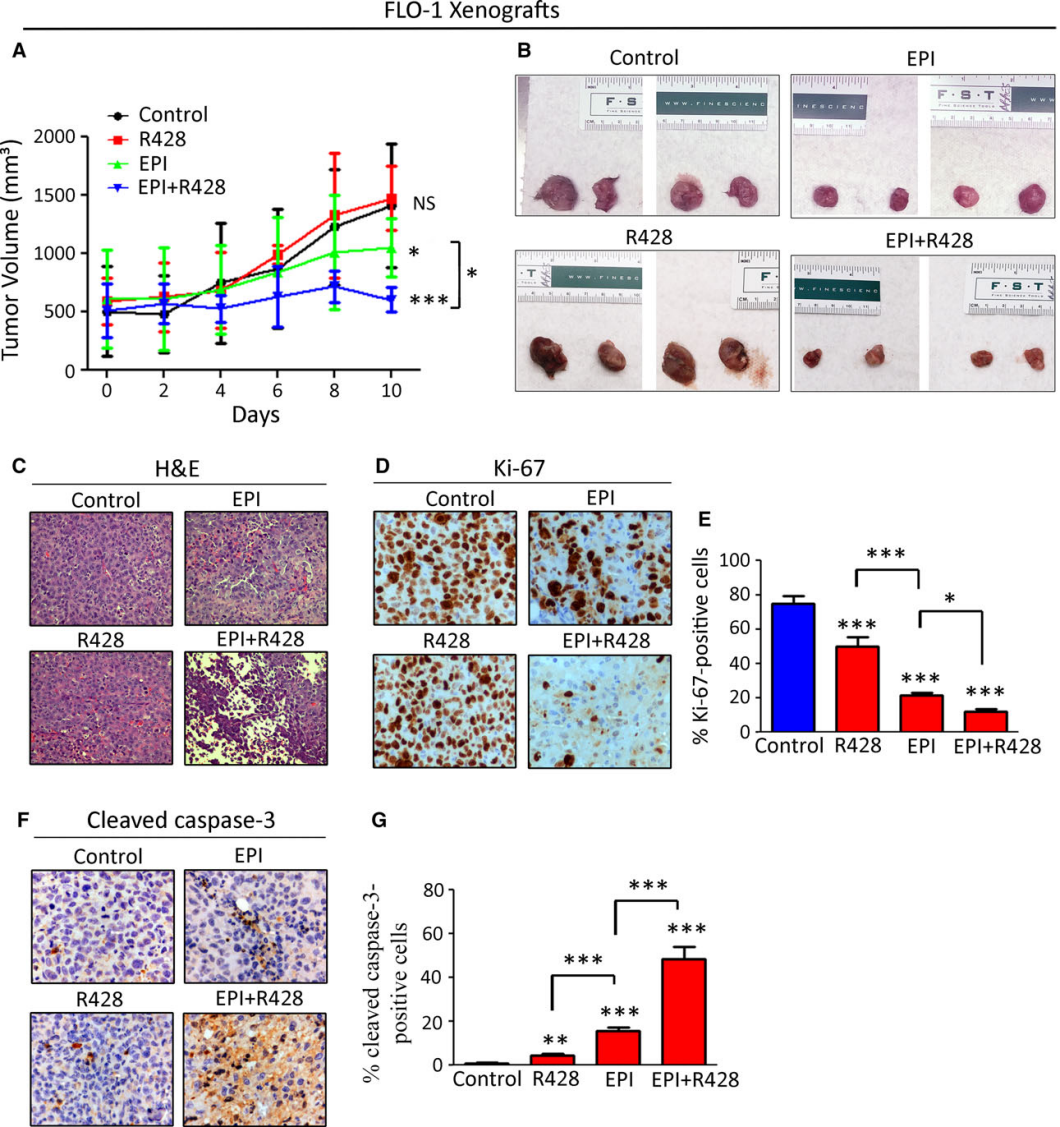
Inhibition of AXL with R428 sensitizes tumors to epirubicin in a xenograft mouse model. (A, B) Rag2 mice were injected with FLO‐1 cells, and the xenografted tumors were allowed to grow until they reach 500 mm^3^ in size, then treated with epirubicin (5 mg·kg^−1^), R428 (10 mg·kg^−1^), or their combination for 10 days. R428 in combination with epirubicin significantly decreased tumor size in comparison with control, R428, or epirubicin‐treated groups (*P* < 0.01). (C) Representative images of H&E staining of tumor xenografts following treatments. IHC analysis for expression of Ki‐67, a marker of proliferation (D, E), and cleaved caspase‐3, a marker of apoptosis (F, G), in representative endpoint tumors of all animal groups. Statistical significance was evaluated by one‐way ANOVA followed by the Newman–Keuls *post hoc* test. NS, not significant; *P < 0.05; **P < 0.01; ***P < 0.001.

To investigate whether inhibiting AXL with R428 could provide a clinical advantage in the treatment of recalcitrant tumors with epirubicin, we used a tumor xenograft mouse model of epirubicin‐resistant FLO‐1 cancer cells that were assessed as shown in Fig. 1. Xenografted tumors were allowed to grow and reach 500 mm^3^ before initiation of treatment of mice with drugs. Our results showed that R428 at a low dose (10 mg·kg^−1^), which was selected based on a published study (Holland *et al*., 2010), had no significant effect on tumor growth (Fig. 6A,B), although a significant reduction in p‐AXL (Y779) protein level was observed (*P* < 0.01, Fig. S7). Based on a published report (Kelly *et al*., 2010) and due to its high toxicity, epirubicin (5 mg·kg^−1^) was used to treat mice for only 10 days. Tumors treated with epirubicin alone continued to grow, but at a significantly slower rate than nontreated control tumors, confirming the resistant phenotype to epirubicin (*P* < 0.05, Fig. 6A,B). In contrast, the combination treatment with epirubicin and R428 significantly blocked tumor growth relative to epirubicin alone (*P* < 0.05, Fig. 6A,B). H&E staining of tumor xenografts clearly revealed more substantial death of tumor tissues following the combination treatment with epirubicin and R428 than single treatments (Fig. 6C). Consistent with these results, IHC analysis of tumor xenografts indicated that the combination treatment with epirubicin and R428 induced the lowest Ki‐67 (Fig. 6D,E) and the highest cleaved caspase‐3 (Fig. 6F,G) protein levels relative to single treatments with either drugs. In line with the growth rate data (Fig. 6A), treatment with a low dose of R428 (10 mg·kg^−1^) alone induced a small decrease in tumor cell proliferation (*P* < 0.001, Fig. 6D,E) and a negligible increase in apoptosis (*P* < 0.01, Fig. 6F,G) relative to control group. Although less effective than the combination treatment, epirubicin (5 mg·kg^−1^) alone decreased tumor proliferation (*P* < 0.001, Fig. 6D,E) and increased apoptosis (*P* < 0.001, Fig. 6F,G) in comparison with control group. Collectively, the results demonstrated that the effect of the combination treatment with R428 and epirubicin was synergistic because it was greater than the sum of effects of both drugs separately.

## 4 Discussion

A comprehensive understanding of the molecular mechanisms underlying chemotherapy drug resistance may pave the way for developing a better clinical management and more effective treatments for cancer patients. Epirubicin, which is a chemotherapy drug in the anthracycline class, is widely used in the treatment of upper gastrointestinal adenocarcinoma (Personeni *et al*., 2016; Pluschnig *et al*., 2013). However, resistance to epirubicin remains a challenging obstacle to a successful treatment of cancer patients. In this study, we have identified a novel role for AXL in promoting resistance to epirubicin in EAC. We confirmed that overexpression of exogenous AXL promotes epirubicin resistance in sensitive EAC cells. Conversely, knockdown or pharmacologic inhibition of endogenous AXL sensitizes resistant EAC cells to epirubicin.

Several recent studies have attempted to identify the underlying mechanisms of epirubicin resistance in gastric (Zhao *et al*., 2009) and breast (Khongkow *et al*., 2013; Nair *et al*., 2016) cancers. Based on a genomewide screen using a small interfering RNA library on gastric cancer cells, a report indicated that loss of function of the putative tumor suppressor gene *GAS1* (growth arrest‐specific 1) was associated with a marked increase in epirubicin resistance (Zhao *et al*., 2009). Previous reports have shown that c‐MYC transcription factor promotes resistance to chemotherapeutics by regulating the stemness of self‐renewing cancer stem cells (Dang, 2012; Zhang *et al*., 2015). Additionally, based on the analysis of the publically available RNAseq data from the Cancer Genome Atlas (TCGA), we found that *c‐MYC* was overexpressed in approximately 68% of the analyzed EAC tumors. This finding is consistent with a previous report of highly frequent *c‐MYC* gene amplification (83.3%) in EAC (von Rahden *et al*., 2006). Unlike *c‐MYC*,* AXL* mRNA overexpression was only detected in approximately 27% of the analyzed EAC tumors (Fig. S8). However, we previously reported overexpression of AXL protein in approximately 52% of EAC tumor samples (Hong *et al*., 2013). This suggests that AXL overexpression is driven in part by transcriptional upregulation in addition to a potential post‐translational mechanism. Based on these findings and the possible functional connection between AXL and c‐MYC, we investigated whether AXL‐dependent epirubicin resistance involves regulation of c‐MYC expression in EAC cells. Indeed, we found that pharmacologic inhibition or genetic knockdown of c‐MYC markedly sensitized resistant cells to epirubicin, confirming that c‐MYC mediates resistance to epirubicin in EAC cells. We next demonstrated that the overexpression of AXL in sensitive EAC cells upregulates the transcriptional activity in addition to mRNA and protein expression of c‐MYC. Conversely, the knockdown of endogenous AXL expression in resistant EAC cells reversed these effects.

AXL has been reported to mediate EMT and a mesenchymal cell phenotype, which confers resistance to several anticancer therapies (Byers *et al*., 2013; Wilson *et al*., 2014; Wu *et al*., 2014; Zhang *et al*., 2012). In early studies, we have demonstrated that AXL mediates TRAIL resistance through regulation of the death‐inducing signal complex (DISC) in EAC cells (Hong and Belkhiri, 2013). We also showed that AXL promotes cisplatin resistance through disruption of c‐ABL/p73β protein interaction in response to cisplatin in EAC cells (Hong *et al*., 2013). In this study, we established that AXL promotes epirubicin resistance through regulation of c‐MYC expression in EAC cells. We demonstrated that AXL transcriptionally upregulates c‐MYC expression through activation of the AKT/β‐catenin pathway without regulating c‐MYC mRNA or protein stability. The AXL‐dependent epirubicin resistance mediated by c‐MYC is likely achieved through regulation of the stemness of a subset of self‐renewing EAC stem cells. Our findings are consistent with the recent report showing that Gas6/AXL signaling stabilizes β‐catenin protein in an AKT‐dependent mechanism, thereby regulating self‐renewal of drug‐resistant leukemia stem cells (Jin *et al*., 2016). We confirmed that pharmacologic inhibition of AXL by R428 is sufficient to down‐regulate c‐MYC expression and overcome epirubicin resistance in EAC cells.

Using an *in vivo* tumor xenograft mouse model, we demonstrated that targeting AXL with a low dose of R428 effectively sensitizes resistant cancer cells to epirubicin. Even though epirubicin‐treated tumors grew at a slower rate relative to control tumors, the tumors treated with epirubicin in combination with R428 almost ceased to grow and significantly decreased in size. The data indicate that the combination treatment is synergistic and strongly suggest that R428 could significantly improve clinical response to epirubicin in EAC patients.

## 5 Conclusions

In this study, we have demonstrated that AXL‐dependent resistance to epirubicin is mediated by c‐MYC in EAC. Mechanistic investigations indicated that AXL transcriptionally upregulates c‐MYC expression through activation of the AKT/β‐catenin pathway in EAC cells. We established that pharmacological inhibition of AXL sensitizes tumors to epirubicin in a xenograft mouse model. Our data indicate that AXL may be a valuable molecular target that could be exploited in the treatment of patients with refractory tumors and support future clinical studies to evaluate the therapeutic potential of AXL inhibitor, R428, in EAC with overexpression of AXL and c‐MYC activation.

## Author contributions

JH designed and performed *in vitro* and *in vivo* experiments. JH and SM analyzed and interpreted the data and contributed to the writing of the manuscript. AB conceived and supervised the project and wrote the manuscript. All authors reviewed the manuscript.

## Supporting information


**Fig. S1.** AXL protein expression and sensitivity to epirubicin in esophageal adenocarcinoma cell lines.

**Fig. S2.** AXL promotes survival of EAC cells in response to epirubicin treatment.

**Fig. S3.** Evaluation of c‐MYC mRNA expression in EAC primary tumors.

**Fig. S4.** Reconstitution of c‐MYC expression restores epirubicin resistance in AXL knockdown cells.

**Fig. S5.** Modulation of AXL expression in esophageal adenocarcinoma cell lines has no significant effect on c‐MYC mRNA or protein stability.

**Fig. S6.** Inhibition of AKT sensitizes EAC cells to epirubicin.

**Fig. S7.** R428 effectively inhibits AXL kinase activity *in vivo*.

**Fig. S8.** Evaluation of AXL mRNA expression in EAC primary tumors.

**Table S1.** List of quantitative real‐time PCR primers sequences.
