# Supplementary figures and images for: Transcriptional upregulation of c‐MYC by AXL confers epirubicin resistance in esophageal adenocarcinoma
Source: Mol Oncol. 2018 Nov 5;12(12):2191–208. doi: 10.1002/1878-0261.12395 (PMC6275285; doi:10.1002/1878-0261.12395)

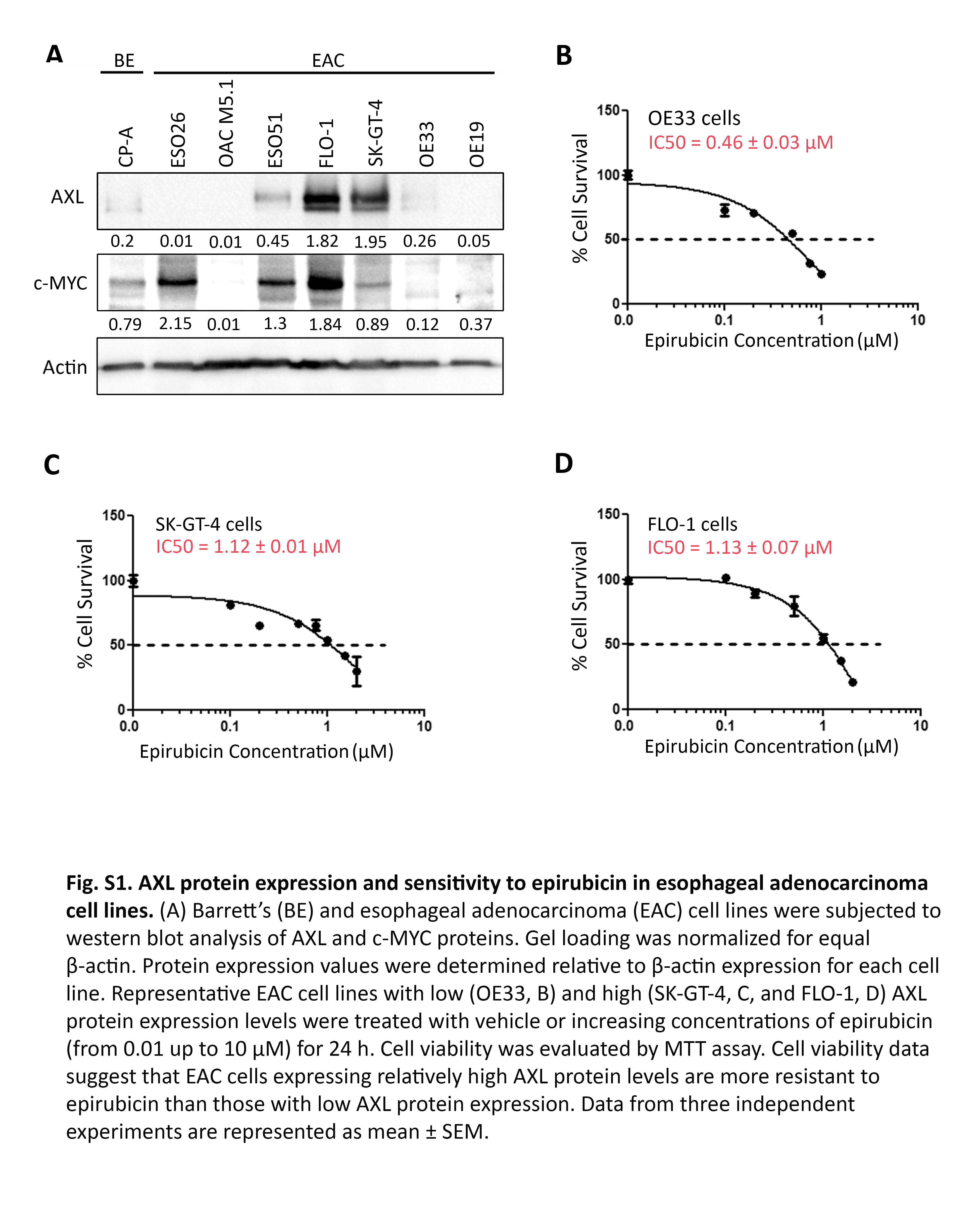

Supplement: Supplementary file 1 — Fig. S1. AXL protein expression and sensitivity to epirubicin in esophageal adenocarcinoma cell lines. [file MOL2-12-2191-s001.tif]

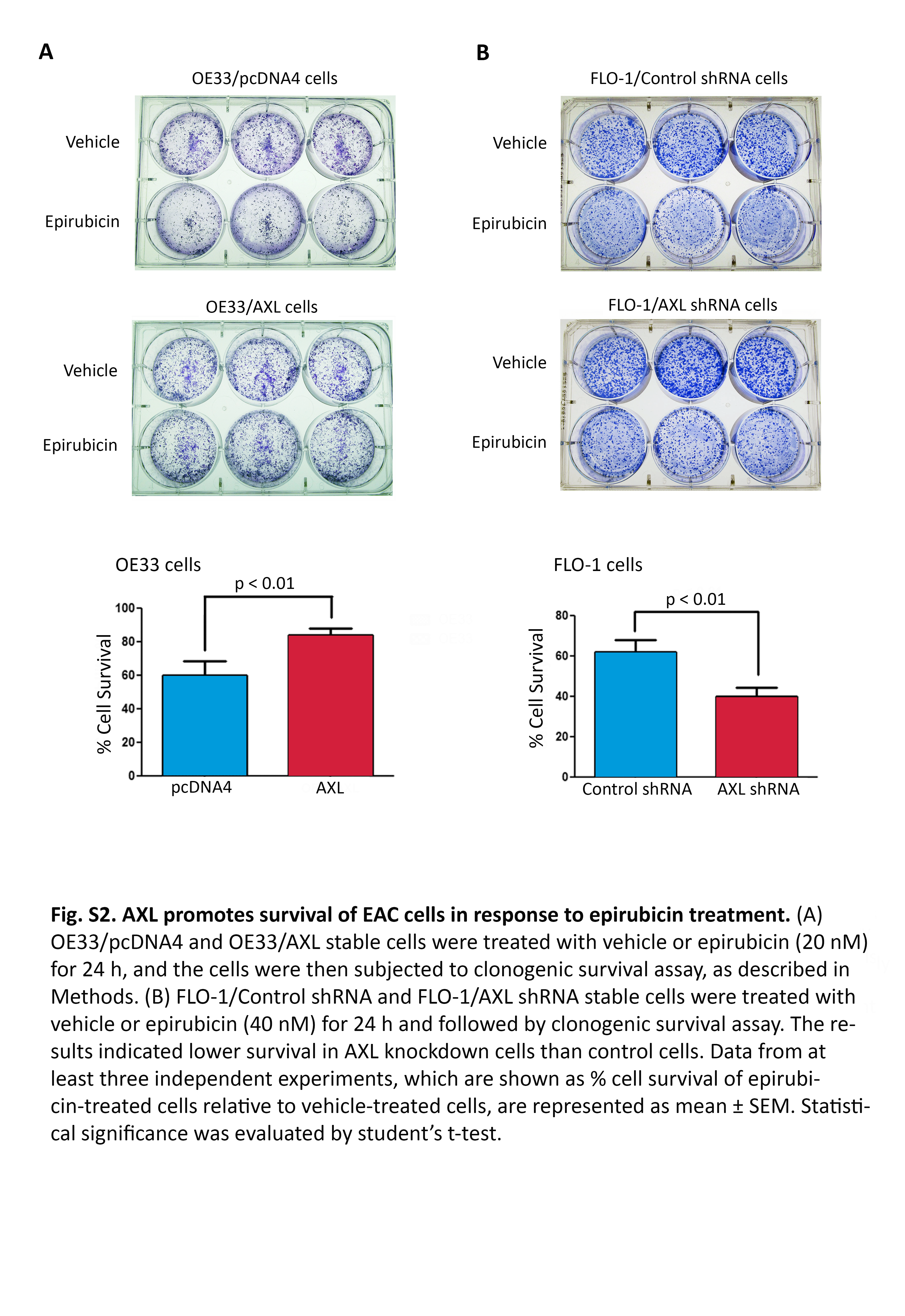

Supplement: Supplementary file 2 — Fig. S2. AXL promotes survival of EAC cells in response to epirubicin treatment. [file MOL2-12-2191-s002.tif]

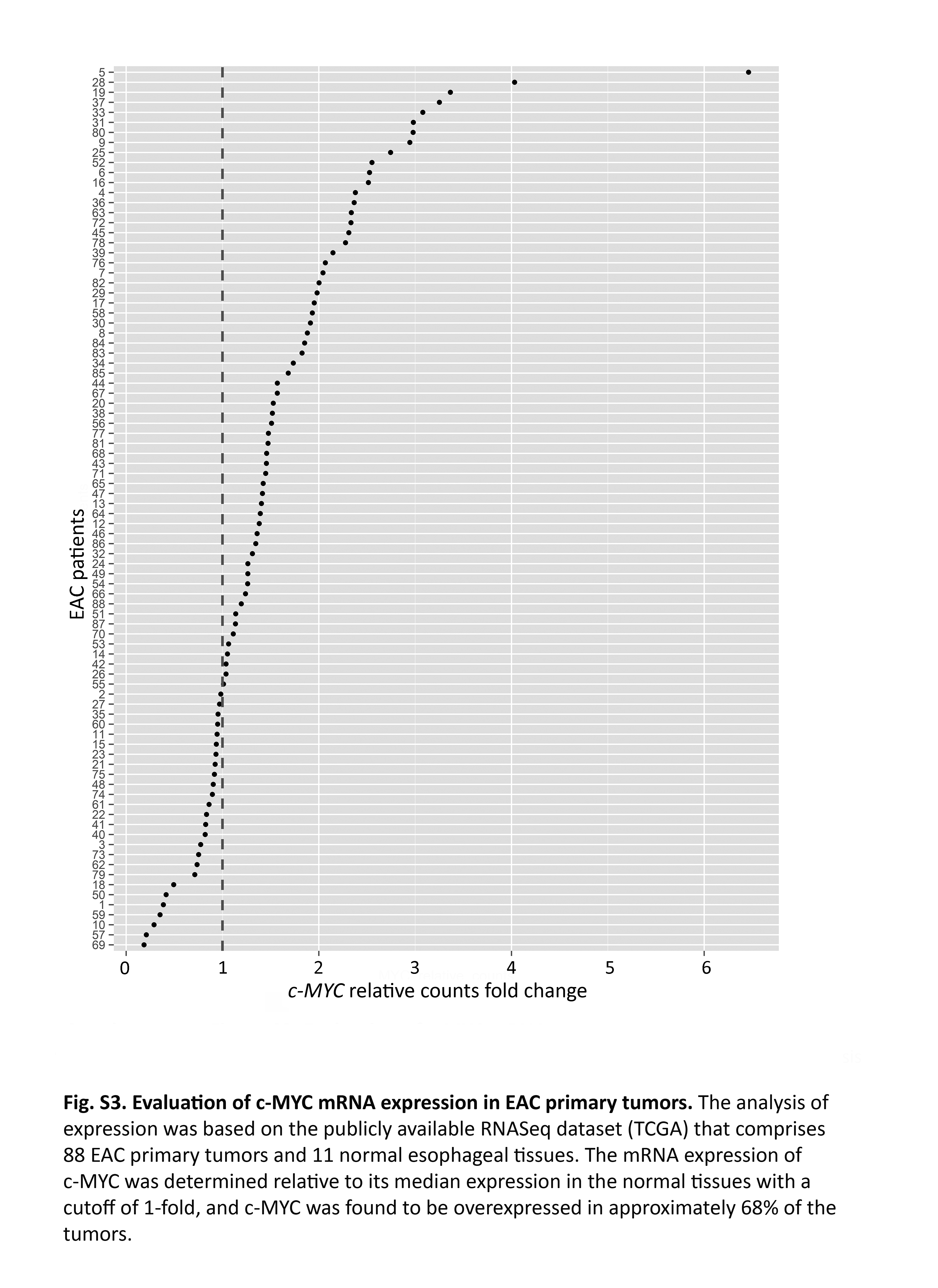

Supplement: Supplementary file 3 — Fig. S3. Evaluation of c‐MYC mRNA expression in EAC primary tumors. [file MOL2-12-2191-s003.tif]

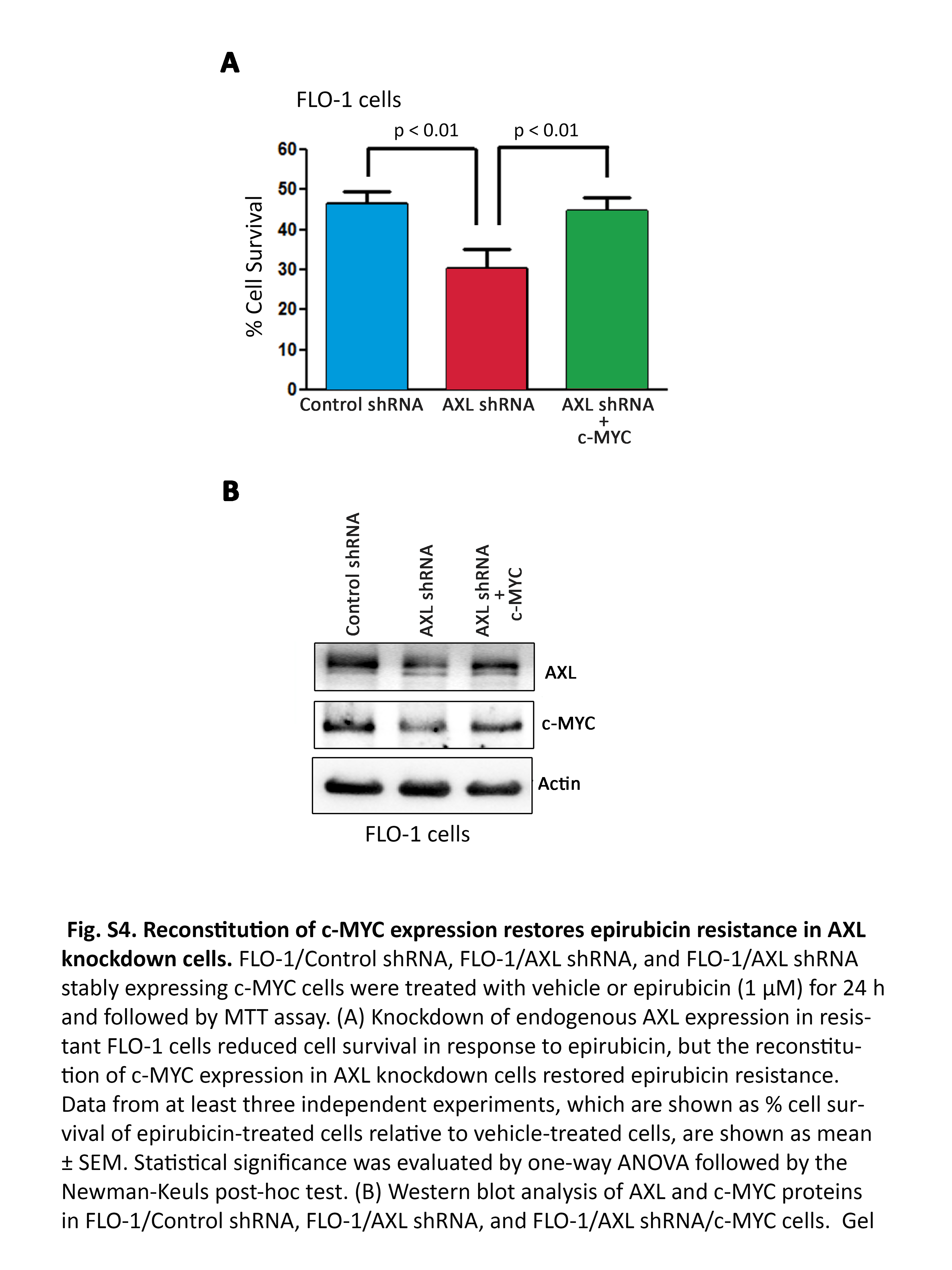

Supplement: Supplementary file 4 — Fig. S4. Reconstitution of c‐MYC expression restores epirubicin resistance in AXL knockdown cells. [file MOL2-12-2191-s004.tif]

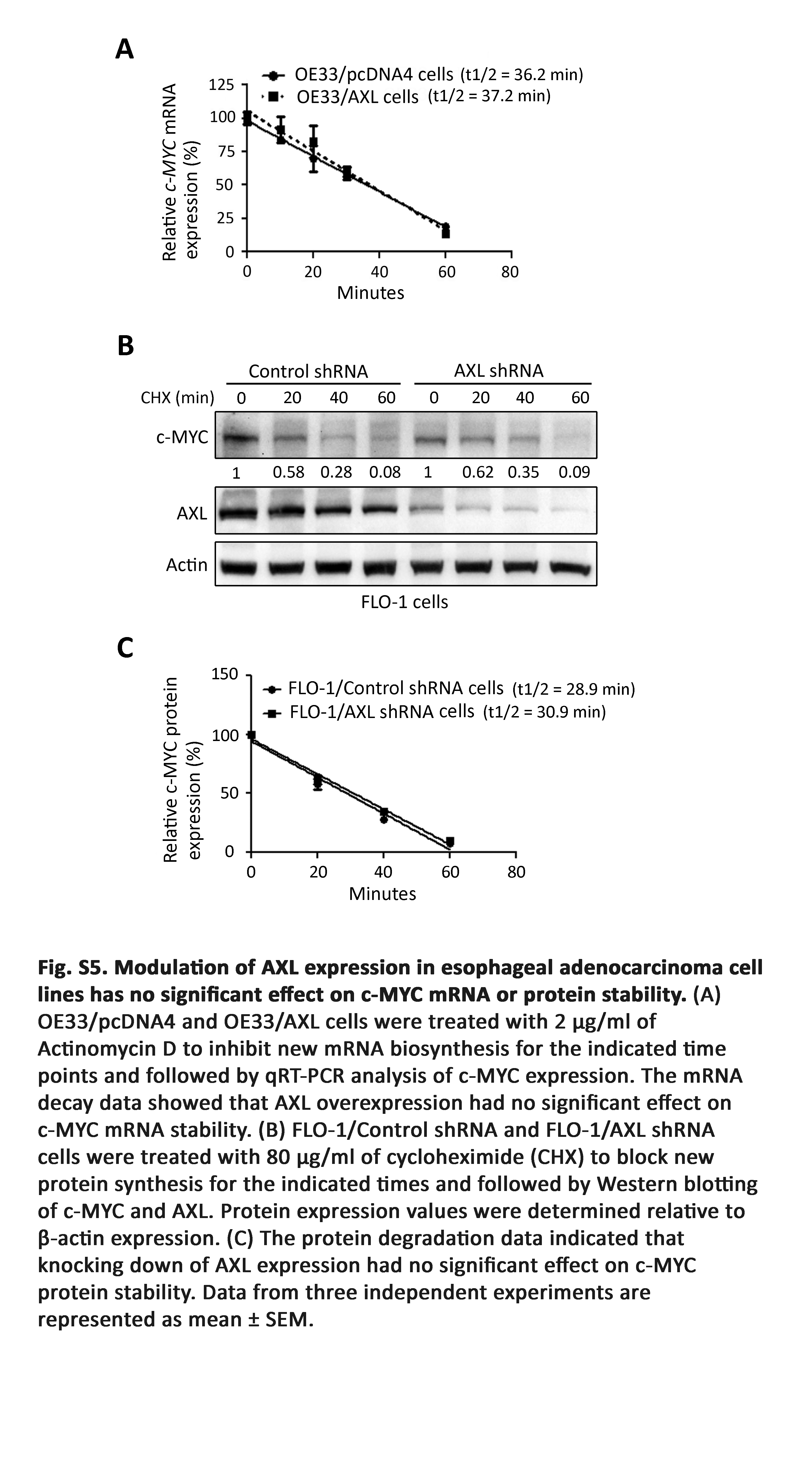

Supplement: Supplementary file 5 — Fig. S5. Modulation of AXL expression in esophageal adenocarcinoma cell lines has no significant effect on c‐MYC mRNA or protein stability. [file MOL2-12-2191-s005.tif]

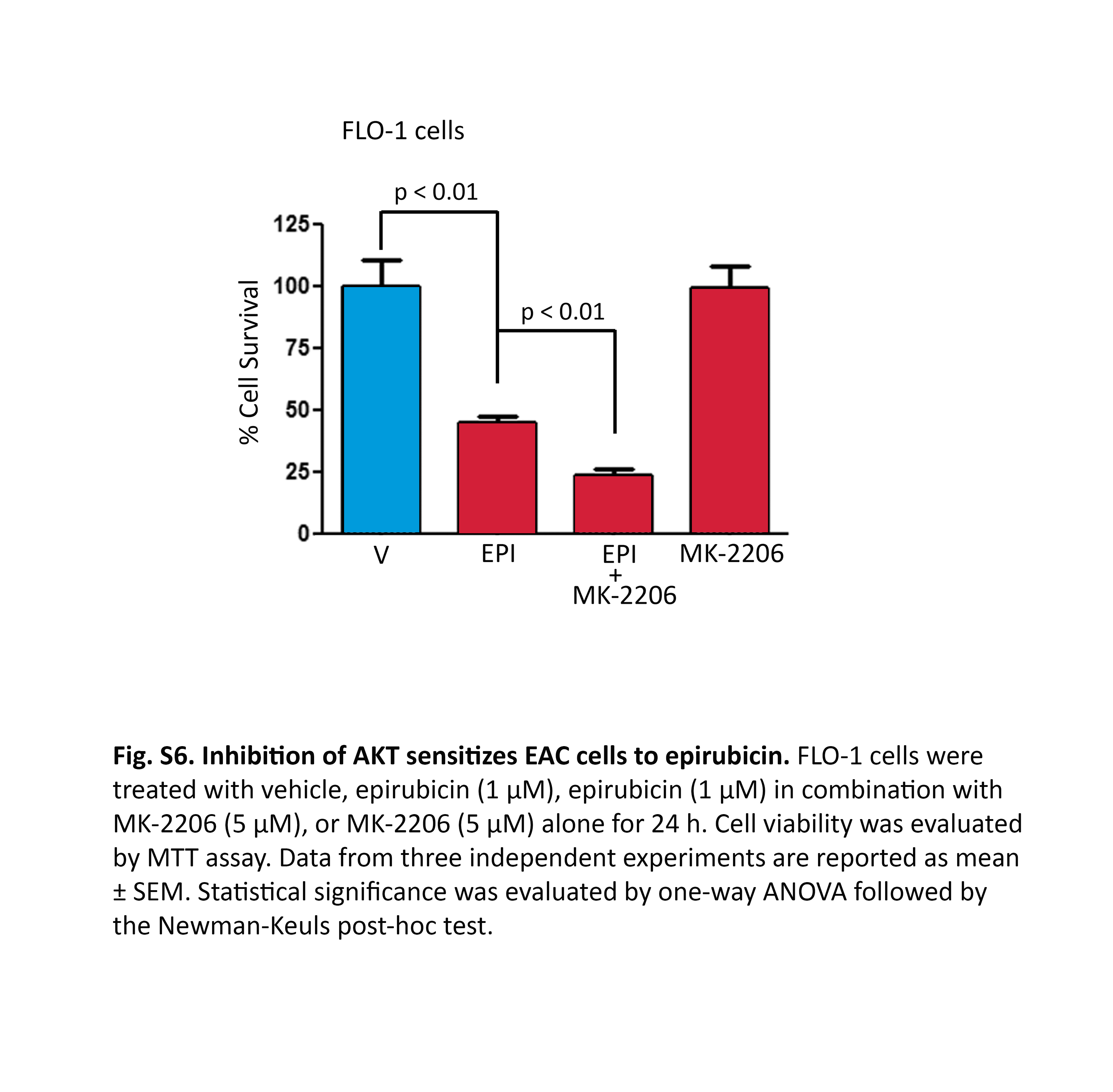

Supplement: Supplementary file 6 — Fig. S6. Inhibition of AKT sensitizes EAC cells to epirubicin. [file MOL2-12-2191-s006.tif]

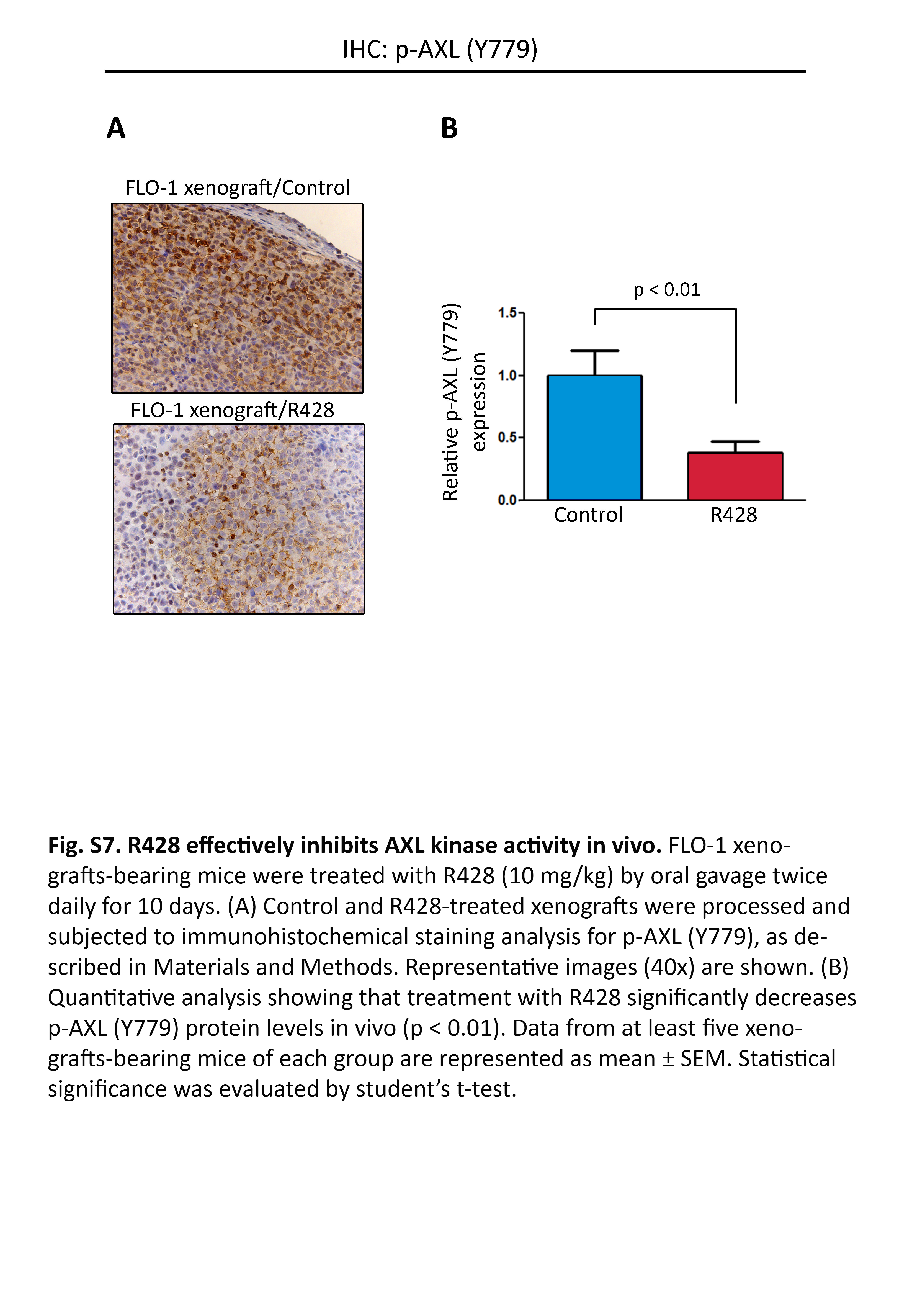

Supplement: Supplementary file 7 — Fig. S7. R428 effectively inhibits AXL kinase activity in vivo. [file MOL2-12-2191-s007.tif]

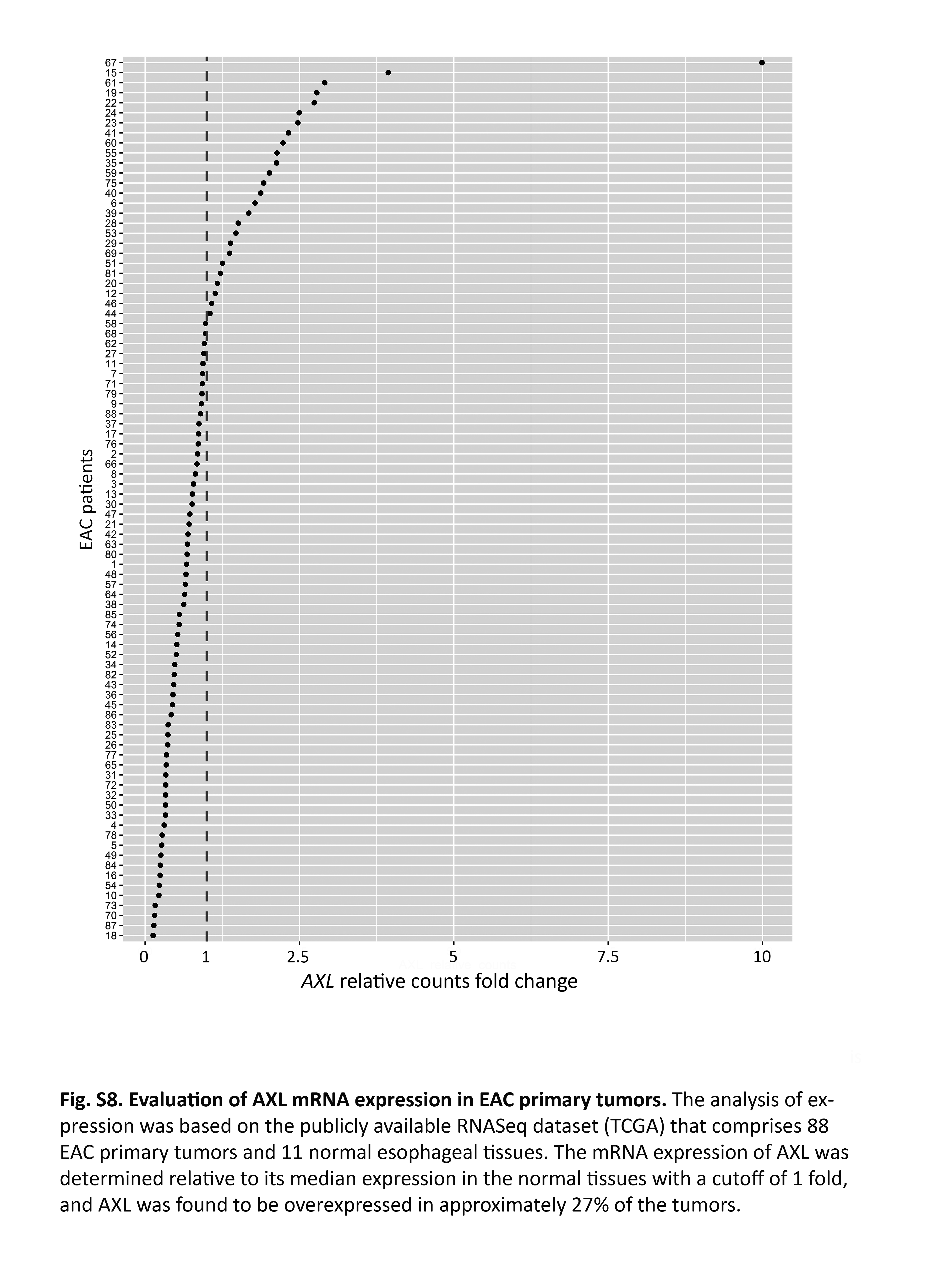

Supplement: Supplementary file 8 — Fig. S8. Evaluation of AXL mRNA expression in EAC primary tumors. [file MOL2-12-2191-s008.tif]
