# Supplementary material for: Transcriptional upregulation of c‐MYC by AXL confers epirubicin resistance in esophageal adenocarcinoma
Source: Mol Oncol. 2018 Nov 5;12(12):2191–208. doi: 10.1002/1878-0261.12395 (PMC6275285; doi:10.1002/1878-0261.12395)
Supplement: Supplementary file 9 — Table S1. List of quantitative real‐time PCR primers sequences. [file MOL2-12-2191-s009.docx]

**Table S1. List of quantitative real-time PCR primers sequences**.

| *HPRT1 (forward)* | 5’-ACCCTTTCCAAATCCTCAGC-3’ |
| --- | --- |
| *HPRT1 (reverse)* | 5’-GTTATGGCGACCCGCAG-3’ |
| *AXL (forward)* | 5’-GAAGGTACCATGACAACCCAGGCAAAGTG-3’ |
| *AXL (reverse)* | 5’-GAACTCGAGACGCCATGGGTGCCAAAC-3’ |
| *c-MYC (forward)* | 5’-CACCGAGTCGTAGTCGAGGT-3’ |
| *c-MYC (reverse)* | 5’-TTTCGGGTAGTGGAAAACCA-3’ |
| *β-actin (forward)* | 5’-CTACAATGAGCTGCGTGTGG-3’ |
| *β-actin (reverse)* | 5’-CTGGGGTGTTGAAGGTCTCA-3’ |
