# Supplementary material for: Transcriptional upregulation of c‐MYC by AXL confers epirubicin resistance in esophageal adenocarcinoma
Source: Mol Oncol. 2018 Nov 5;12(12):2191–208. doi: 10.1002/1878-0261.12395 (PMC6275285; doi:10.1002/1878-0261.12395)
Supplement: Supplementary file 10 [file MOL2-12-2191-s010.docx]

**Supplementary Figure Legends**

**Fig. S1. AXL protein expression and sensitivity to epirubicin in esophageal adenocarcinoma cell lines.**

(A) Barrett’s (BE) and esophageal adenocarcinoma (EAC) cell lines were subjected to western blot analysis of AXL and c-MYC proteins. Gel loading was normalized for equal β-actin. Protein expression values were determined relative to β-actin expression for each cell line. Representative EAC cell lines with low (OE33, B) and high (SK-GT-4, C, and FLO-1, D) AXL protein expression levels were treated with vehicle or increasing concentrations of epirubicin (from 0.01 up to 10 µM) for 24 h. Cell viability was evaluated by MTT assay. Cell viability data suggest that EAC cells expressing relatively high AXL protein levels are more resistant to epirubicin than those with low AXL protein expression. Data from three independent experiments are represented as mean ± SEM.

**Fig. S2. AXL promotes survival of EAC cells in response to epirubicin treatment.**

(A) OE33/pcDNA4 and OE33/AXL stable cells were treated with vehicle or epirubicin (20 nM) for 24 h, and the cells were then subjected to clonogenic survival assay, as described in Supplementary Methods. (B) FLO-1/Control shRNA and FLO-1/AXL shRNA stable cells were treated with vehicle or epirubicin (40 nM) for 24 h and followed by clonogenic survival assay. The results indicated lower survival in AXL knockdown cells than control cells. Data from at least three independent experiments, which are shown as % cell survival of epirubicin-treated cells relative to vehicle-treated cells, are represented as mean ± SEM. Statistical significance was evaluated by student’s t-test.

**Fig. S3. Evaluation of c-MYC mRNA expression in EAC primary tumors.**

The analysis of expression was based on the publicly available RNASeq dataset (TCGA) that comprises 88 EAC primary tumors and 11 normal esophageal tissues. The mRNA expression of c-MYC was determined relative to its median expression in the normal tissues with a cutoff of 1-fold, and c-MYC was found to be overexpressed in approximately 68% of the tumors.

**Fig. S4. Reconstitution of c-MYC expression restores epirubicin resistance in AXL knockdown cells.**

FLO-1/Control shRNA, FLO-1/AXL shRNA, and FLO-1/AXL shRNA stably expressing c-MYC cells were treated with vehicle or epirubicin (1 µM) for 24 h and followed by MTT assay. (A) Knockdown of endogenous AXL expression in resistant FLO-1 cells reduced cell survival in response to epirubicin, but the reconstitution of c-MYC expression in AXL knockdown cells restored epirubicin resistance. Data from at least three independent experiments, which are shown as % cell survival of epirubicin-treated cells relative to vehicle-treated cells, are shown as mean ± SEM. Statistical significance was evaluated by one-way ANOVA followed by the Newman-Keuls post-hoc test. (B) Western blot analysis of AXL and c-MYC proteins in FLO-1/Control shRNA, FLO-1/AXL shRNA, and FLO-1/AXL shRNA/c-MYC cells. Gel loading was normalized for equal β-actin.

**Fig. S5. Modulation of AXL expression in esophageal adenocarcinoma cell lines has no significant effect on c-MYC mRNA or protein stability.**

(A) OE33/pcDNA4 and OE33/AXL cells were treated with 2 µg/ml of Actinomycin D to inhibit new mRNA biosynthesis for the indicated time points and followed by qRT-PCR analysis of c-MYC expression. The mRNA decay data showed that AXL overexpression had no significant effect on c-MYC mRNA stability. (B) FLO-1/Control shRNA and FLO-1/AXL shRNA cells were treated with 80 µg/ml of cycloheximide (CHX) to block new protein synthesis for the indicated times and followed by Western blotting of c-MYC and AXL. Protein expression values were determined relative to β-actin expression. (C) The protein degradation data indicated that knocking down of AXL expression had no significant effect on c-MYC protein stability. Data from three independent experiments are represented as mean ± SEM.

**Fig. S6. Inhibition of AKT sensitizes EAC cells to epirubicin.**

FLO-1 cells were treated with vehicle, epirubicin (1 µM), epirubicin (1 µM) in combination with MK-2206 (5 µM), or MK-2206 (5 µM) alone for 24 h. Cell viability was evaluated by MTT assay. Data from three independent experiments are reported as mean ± SEM. Statistical significance was evaluated by one-way ANOVA followed by the Newman-Keuls post-hoc test.

**Fig. S7. R428 effectively inhibits AXL kinase activity *in vivo*.**

FLO-1 xenografts-bearing mice were treated with R428 (10 mg/kg) by oral gavage twice daily for 10 days. (A) Control and R428-treated xenografts were processed and subjected to immunohistochemical staining analysis for p-AXL (Y779), as described in Materials and Methods. Representative images (40x) are shown. (B) Quantitative analysis showing that treatment with R428 significantly decreases p-AXL (Y779) protein levels *in vivo* (p < 0.01). Data from at least five xenografts-bearing mice of each group are represented as mean ± SEM. Statistical significance was evaluated by student’s t-test.

**Fig. S8. Evaluation of AXL mRNA expression in EAC primary tumors.**

The analysis of expression was based on the publicly available RNASeq dataset (TCGA) that comprises 88 EAC primary tumors and 11 normal esophageal tissues. The mRNA expression of AXL was determined relative to its median expression in the normal tissues with a cutoff of 1 fold, and AXL was found to be overexpressed in approximately 27% of the tumors.
